# Economic costs and health utility values associated with extremely preterm birth: Evidence from the EPICure2 cohort study

**DOI:** 10.1111/ppe.12906

**Published:** 2022-07-13

**Authors:** Felix Achana, Samantha Johnson, Yanyan Ni, Neil Marlow, Dieter Wolke, Kamran Khan, Stavros Petrou

**Affiliations:** ^1^ Nuffield Department of Primary Care Health Sciences University of Oxford Oxford UK; ^2^ Division of Health Sciences, Warwick Medical School University of Warwick Coventry UK; ^3^ Department of Health Sciences University of Leicester Leicester UK; ^4^ Elizabeth Garrett Anderson Institute for Women's Health University College London London UK; ^5^ Department of Psychology University of Warwick Coventry UK; ^6^ Warwick Clinical Trials Unit University of Warwick Coventry UK

**Keywords:** costs, economic, extremely preterm, health utilities

## Abstract

**Background:**

Preterm birth is associated with adverse health and developmental sequelae that impose a burden on finite resources and significant challenges for individuals, families and societies.

**Objectives:**

To estimate economic outcomes at age 11 associated with extremely preterm birth using evidence from a whole population study (EPICure2 study).

**Methods:**

The study population comprised a sample of children born at ≤26 completed weeks of gestation during 2006 in England (*n* = 200) and a comparison group of classmates born at term (*n* = 143). Societal costs were estimated using parent and teacher reports of service utilisation, and valuations of work losses and additional care costs to families. Utility scores for the Health Utilities Index Mark 2 (HUI2) and Mark 3 (HUI3) were generated using UK and Canadian value sets. Generalised linear regression was used to estimate the impact of extremely preterm birth on societal costs and utility scores.

**Results:**

Unadjusted mean societal costs that excluded provision of special educational support in mainstream schools during the 11th year after birth were £6536 for the extremely preterm group and £3275 for their classmates, generating a difference of £3262 (95% confidence interval [CI] £1912, £5543). The mean adjusted cost difference was £2916 (95% CI £1609, £4224), including special educational needs provision in mainstream schools increased the adjusted cost difference to £4772 (95% CI £3166, £6378). Compared with birth at term, extremely preterm birth generated mean‐adjusted utility decrements ranging from 0.13 (95% CI 0.09, 0.18) based on the UK HUI2 statistical inference tariff to 0.28 (95% CI 0.18, 0.37) based on the Canadian HUI3 tariff.

**Conclusions:**

The adverse economic impact of extremely preterm birth persists into late childhood. Further longitudinal studies conducted from multiple perspectives are needed to understand the magnitude, trajectory and underpinning mechanisms of economic outcomes following extremely preterm birth.

**Synopsis**

**Study question**

What are the economic consequences of extremely preterm birth for individuals, families and society during the early to late childhood years?

**What is already known**

We previously showed that extremely preterm birth is associated with increased public sector costs by £2477 (2006–2007 prices) and a Health Utilities Index Mark 3 (HUI3) utility decrement of 0.31, on average, at age 11.

**What this study adds**

We have expanded our previous estimates of the economic effects of extremely preterm birth in early to late childhood to capture societal costs such as lost productivity and additional care costs to families attributable to the child's health.

## 1 BACKGROUND

The adverse health and developmental sequelae arising from preterm birth impose a burden on finite resources and significant challenges for health services, individuals, families and societies.^1^, ^2^ Compared with term‐born babies, preterm babies are more likely to have neurodevelopmental impairments, such as cerebral palsy, visual disorders, cognitive deficits and learning difficulties, which impact on long‐term physical health and development.^3^ These challenges extend beyond childhood into adolescence and adulthood.^4^

The rate of live preterm births in England and Wales has ranged between 7% and 8% since 2010.^5^ Given the inverse association between gestational age at birth and developmental sequelae, this subgroup of infants is at greater risk of adverse outcomes in both early and middle childhood.^6^, ^7^ Evidence from both the 1995 and 2006 EPICure cohorts suggests that gestational age at birth was the single most important predictor of survival and neurodevelopmental outcomes for extremely preterm babies born in 2006.^4^ Cerebral palsy was present in 14% of survivors at age three whilst neurodevelopmental impairment decreased with increasing gestational age from 45% for those born at 22–23 completed weeks to 20% at 26 completed weeks gestation.^4^

Economic outcomes associated with extremely preterm birth include economic costs borne by the health services, other sectors of the economy and families, and preference‐based health utility values that act as inputs into cost‐utility analyses.^8^, ^9^, ^10^, ^11^, ^12^, ^13^, ^14^ A systematic literature review suggests that initial hospitalisation costs alone range between $111,152 and $576,972 on average per infant born at 24 weeks' gestation compared with $930 to $7114 on average per infant born at term (2015 US dollar prices).^15^ However, the evidence on the longer‐term economic costs associated with extremely preterm birth was sparse.

In the UK, the prevalence of special educational needs (SEN) in children of school‐going age remains stable from primary to secondary school in which 12.6% and 11.5% of pupils have SEN support, respectively.^16^ These figures suggest SEN support at 11 years could be considered as representative of secondary school outcomes.^16^ Using data from the 1995 EPICure birth cohort, we previously estimated that extremely preterm birth was associated with increased public sector costs of £2477 (2006–7 prices) and a decrement in health utility of 0.312 as measured by the Health Utilities Index Mark 3, on average, during the 11th year. In our most recent analysis of the EPICure2 cohort, we found little to no improvement in the rates of neurodevelopmental impairment or low academic attainment at 11 years compared with the 1995 EPICure birth cohort despite over a decade of improvements in neonatal care and survival.^17^, ^18^ Using data from the EPICure2 cohort, this study aimed to estimate the impact of extremely preterm birth on economic costs for the public sector, families and society, and on health utility values, for these births in 2006.

## 2 METHODS

### 2.1 Study population

The data used in this investigation are drawn from a whole population study of infants born at ≤26 completed weeks of gestation in England in 2006. Of 1031 surviving children born extremely preterm, 576 (56%) were assessed at age 3 years, and a sample of 200 children (19.4%) was evaluated at age 11 years.^17^ Baseline characteristics were comparable between those assessed at year 11 and dropouts with respect to perinatal characteristics, maternal age and Index of Multiple Deprivation (IMD).^17^ The sample for the present analysis comprises 200 extremely preterm children who were assessed at age 11, and a comparison group of 143 classmates selected from mainstream schools who were born at term and matched on age and sex to an extremely preterm child where possible. A detailed description of the EPICure2 study population and comparison of cohort characteristics and outcomes with the previous EPICure cohort has been reported elsewhere.^17^

### 2.2 Estimation of costs

Parents and teachers completed questionnaires about the child's health and utilisation of services over the preceding year (see Appendix S1). Parents also provided details of time‐off work and the additional costs borne by families over the preceding year related to the child's health status. Details of the type of school the child attended (mainstream school, special school or mainstream school with special unit attached) were obtained from the study assessment records. For children attending a mainstream school, teachers provided details of SEN provision, including whether the child had an Education, Health and Care Plan.

Estimates of service provision were expressed in contact hours per year for community health and social care services and contact hours per week for education services. For hospital admissions, estimates of service provision were expressed as patient days with part of a day counted as a 24‐h period. For education services, estimates of service provision reflected the level of educational assistance within each type of educational establishment.

Health and social care services in the community were valued by attaching unit costs to resource inputs.^19^, ^20^, ^21^ Primary care costs were derived from the Personal Social Services Research Unit (PSSRU) Unit Costs of Health and Social Care 2019 edition.^21^ Medication costs were obtained from the prescription cost analysis 2019 database,^20^ electronic searches of the British National Formulary (BNF) 2019 edition^22^ and searches of the literature.^23^, ^24^, ^25^, ^26^ Unit costs for hospital‐based care were obtained from the 2019 National Reference Costs Main schedules^19^ and took into account the clinical specialty and inpatient length of stay. Inpatient length of stays was considered as short‐stays for day‐long admissions and long‐stays for admissions lasting ≥2 days in line with NHS Reference cost calculations.^27^ Unit cost for education services included the cost per year of attending a mainstream primary school, special school or special unit attached to a mainstream school and were obtained from UK government official statistics^28^ and briefing papers.^29^ Work absences as a result of the child's health were valued using national gender‐specific earnings.^30^ All costs expressed in pounds sterling and valued at 2019 prices, or inflated to 2019 values, were appropriate.^21^

### 2.3 Estimation of health utilities

The main parent (usually the mother) completed the 15‐item proxy‐assessed usual health status assessment questionnaire for the Health Utilities Index (HUI), which encompasses both Mark 2 (HUI2) and Mark 3 (HUI3) health status classification systems.^31^ The HUI2 was developed specifically for use with children and covers seven attributes: sensation, mobility, emotion, cognition, self‐care, pain and fertility, each with three to five levels.^31^, ^32^ The HUI3 covers eight attributes: cognition, vision, hearing, speech, ambulation, dexterity, emotion and pain. Function within each attribute is graded on a 5‐ or 6‐point scale ranging from normal function (level 1) to severe impairment (levels 3, 4, 5 or 6). The HUI2 has value sets for the UK and Canada but the HUI3 only has a Canadian value set. We applied UK algorithms^33^ for generating HUI2 utilities and Canadian HUI^31^, ^32^ algorithms to generate values for HUI2 and HUI3 health utilities.

### 2.4 Statistical analysis

Baseline characteristics for the extremely preterm children and their term‐born classmates were summarised in tables as means and standard deviations for continuous variables and percentages for categorical variables. Comparisons of costs associated with each category of resource use and total public sector and total societal costs were made between the extremely preterm children and their classmates, and between prespecified groups of extremely preterm children of varying gestational age at birth and those born at term. Between‐group differences in mean costs were estimated together with 95% confidence intervals (CIs) generated using nonparametric bootstrapping with replacement, based on 1000 replications.^34^

For each of the seven attributes of the HUI2 (and eight attributes for HUI3), we compared the proportion of children with suboptimal levels of function (defined as below level 1) between the comparator groups using Fisher's exact test. Between‐group differences in health utility values for each instrument were generated together with the associated 95% confidence intervals.

Multivariable generalised linear regressions were fitted assuming gamma distribution and a logarithmic link function for costs and utilities. Covariates in the regression equations included age, sex (male, female), marital status (married, single, cohabiting), race (White, Non‐White), Index of Multiple Deprivation score (1st to 4th most deprived vs. 5th to 10th least‐deprived decile) and number of smokers in the household (one or more vs. none) at child aged 11.

Covariates were selected based on clinical and epidemiological relevance and an assessment of whether the observed differences in the distribution of baseline characteristics between the two groups were meaningful. Analyses were performed using statistical package R version 4.0.1.^35^

### 2.5 Missing data

Multiple imputation using chained equations with predictive mean matching was used to predict values for missing costs and utilities, assuming data were missing at random. Fifty imputed datasets were generated at the level of the type of service or resource category stratified by gestational age at birth in line with current best practice recommendations.^36^, ^37^

### 2.6 Ethics approval

Written informed consent was obtained from both children and parents prior to participation. Ethical approval was obtained from both the University College London and the University of Leicester Research Ethics Committees.

## 3 RESULTS

### 3.1 Study population

Baseline characteristics of the study population are summarised in Table 1. A total of 343 children were assessed at age 11 of which 200 (58.3%) were born extremely preterm and 143 (41.7%) born at term. In the extremely preterm group, 15 (7.5%) were born at ≤23 completed weeks of gestation, 28 (14%) at 24 weeks, 69 (34.5%) at 25 weeks and 88 (44%) at 26 weeks. Children in the extremely preterm group were comparable to those born at term in age and sex distribution, parental smoking status and IMD score. Because of the adopted sampling strategy, the extremely preterm group was more likely to be of non‐white ethnicity (32.8% vs. 15.3%) and to receive SEN support (12.5% vs. 0.0%), but less likely to speak English at home (55% vs. 71.3%).

**TABLE 1:** Baseline characteristics of extremely preterm children and their classmates

Characteristic	Extremely preterm	Classmates
	(*n* = 200)	(*n* = 143)
Age (mean (SD)), years	11.83 (0.55)	11.76 (0.61)
% Female	100 (50%)	80 (55.9%)
Parent completing questionnaire		
Mother	155 (77.5%)	110 (76.9%)
Father	12 (6%)	11 (7.7%)
Other	10 (5%)	3 (2.1%)
Unknown	23 (11.5%)	19 (13.3%)
Marital status		
Married	119 (59.5%)	88 (61.5%)
Single	21 (10.5%)	9 (6.3%)
Living with partner	17 (8.5%)	11 (7.7%)
Separated/divorced	13 (6.5%)	17 (11.9%)
Widowed	1 (0.5%)	0 (0.0%)
Unknown	29 (14.5%)	18 (12.6%)
Parental age (years)		
<30	1 (0.5%)	0 (0.0%)
30–39	31 (15.5%)	26 (18.2%)
40–49	100 (50%)	72 (50.3%)
≥50	22 (11%)	15 (10.5%)
Accommodation		
Living with the mother/father	127 (63.5%)	95 (66.4%)
Living with the other partner	10 (5%)	6 (4.2%)
Previously with partner, now alone	21 (10.5%)	21 (14.7%)
Never lived with a partner	4 (2%)	1 (0.7%)
Other	9 (4.5%)	2 (1.4%)
Unknown	29 (14.5%)	18 (12.6%)
Own home		
Council rented	27 (13.5%)	14 (9.8%)
Private rent	13 (6.5%)	12 (8.4%)
Own home or have mortgage	124 (62%)	93 (65%)
Other	7 (3.5%)	4 (2.8%)
Unknown	29 (14.5%)	20 (14.0%)
Highest parental education level		
None	2 (1%)	1 (0.7%)
O‐level/GCSE/Scottish standards	12 (6%)	8 (5.6%)
Vocational/NVQ/CSE	16 (8%)	8 (5.6%)
BTEC diploma/A‐level/Scottish higher	11 (5.5%)	9 (6.3%)
Diploma or HND	14 (7%)	10 (7.0%)
Unknown	79 (39.5%)	53 (37.1%)
University degree	34 (17%)	29 (20.3%)
Postgraduate qualification	32 (16%)	25 (17.5%)
Employment status, parent		
Unemployed	8 (4%)	3 (2.1%)
Full‐time student	0 (0%)	3 (2.1%)
Employed	108 (54%)	80 (55.9%)
Self‐employed	23 (11.5%)	17 (11.9%)
Homemaker	20 (10%)	13 (9.1%)
Retired	2 (1%)	1 (0.7%)
Other	10 (5%)	6 (4.2%)
Unknown	29 (14.5%)	20 (14.0%)
Index of multiple deprivation		
1	19 (9.5%)	8 (5.6%)
2	21 (10.5%)	19 (13.3%)
3	30 (15%)	23 (16.1%)
4	19 (9.5%)	15 (10.5%)
≥5	106 (53%)	73 (51%)
Unknown	5 (2.5%)	5 (3.5%)
Language spoken at home		
English only	110 (55%)	102 (71.3%)
Mostly english	49 (24.5%)	16 (11.2%)
Only other	2 (1%)	1 (0.7%)
Mostly other	10 (5%)	6 (4.2%)
Unknown	29 (14.5%)	18 (12.6%)
Ethnicity of child		
Asian	24 (12%)	11 (7.7%)
Black	20 (10%)	4 (2.8%)
Mixed	15 (7.5%)	4 (2.8%)
White	101 (50.5%)	98 (68.5%)
Other	6 (3%)	3 (2.1%)
Unknown	34 (17%)	23 (16.1%)
Number of smokers in household		
0	113 (56.5%)	78 (54.5%)
1	17 (8.5%)	15 (10.5%)
2	5 (2.5%)	5 (3.5%)
Unknown	65 (32.5%)	45 (31.5%)
Smoking during pregnancy		
No	154 (77%)	114 (79.7%)
Yes	14 (7%)	10 (7.0%)
Unknown	32 (16%)	19 (13.3%)
School year		
Primary school	57 (28.5%)	53 (37.1%)
Secondary school	86 (43%)	61 (42.7%)
Unknown	57 (28.5%)	29 (20.3%)
Type of school		
Mainstream	173 (86.5%)	142 (99.3%)
SEN unit attached to mainstream school	3 (1.5%)	0 (0%)
SEN	22 (11%)	0 (0%)
Unknown	2 (1%)	1 (0.7%)
EHC plan		
No	101 (50.5%)	115 (80.4%)
Yes	48 (24%)	0 (0%)
Unknown	51 (25.5%)	28 (19.6%)
Gestational age (weeks)		
23	15 (7.5%)	0 (0.0%)
24	28 (14%)	0 (0.0%)
25	69 (34.5%)	0 (0.0%)
26	88 (44%)	0 (0.0%)
Term	0 (0%)	143 (100%)

Resource use questionnaire completion rates varied by the respondent, with greater completion rates for parent questionnaires (range between 73% and 94% of sample) than teacher questionnaires (range between 68% and 78%). As a result, only 221 (64%) of the 343 children making up the total study sample (preterm: 119 [53.8%]; term: 102 [46.2%]) had complete utilisation data across all categories of health, social care and education services. For health utility outcomes, 161 (81%) children in the extremely preterm group and 120 (84%) of their classmates had complete data for calculating HUI2 and HUI3 utility scores.

### 3.2 Service utilisation and costs

Utilisation rates were, on average, higher in the extremely preterm group than for their classmates (Table S1). Table 2 presents the main cost comparisons. The evidence suggested that extremely preterm birth was associated with higher costs compared with birth at term for all categories of public sector costs, additional care costs to families and values of work absences included in our total societal cost calculations. Based on the 221 children with complete service utilisation data, the mean total societal costs (excluding the cost of SEN provision in mainstream schools) over the 12‐month period was £6536 for the extremely preterm group and £3275 for their classmates, generating an unadjusted mean cost difference of £3262 (95% confidence interval [CI]: £1936, £5288). The adjusted mean cost difference was £2916 (95% CI £1609, £4224) based on a complete case analysis and £4081 (95% CI £2814, £5349) using multiple imputations to account for missing data. When the analysis was stratified by gestational age (Table S2), compared with birth at term, extremely preterm birth increased total societal costs across all gestational age categories.

**TABLE 2:** Mean costs and mean cost differences stratified by cost category (UK pound sterling, 2019 prices)

Cost category	Mean costs (95%CI)^a^, £		Mean cost difference (95% CI), £		
	Preterm group (*N* = 119)	Classmates (*N* = 102)	Unadjusted complete cases	Adjusted complete cases	Adjusted multiple imputation
Hospital inpatient care costs	71 (0, 394)	0^b^	71 (0, 394)		
Hospital outpatient, A&E and day care costs	290 (216, 388)	137 (80, 207)	153 (48, 258)		
Total hospital costs	361 (244, 591)	137 (85, 206)	224 (92, 454)		
Total community care costs	331 (250, 457)	151 (126, 213)	181 (90, 312)	332 (145, 519)	554 (292, 816)
Total medication costs	503 (140, 2285)	27 (6, 105)	476 (114, 2271)	927 (−172, 2027)	676 (−211, 1563)
Total health and social care costs	1195 (777, 2899)	315 (242, 427)	881 (464, 2545)	1635 (554, 2715)	1479 (834, 2124)
Total additional care costs	69 (28, 142)	2 (0, 12)	66 (25, 141)	331 (16, 645)	457 (−262, 1175)
Indirect costs associated with lost productivity	484 (156, 1332)	69 (40, 122)	414 (106, 1286)	160 (−139, 459)	2371 (−92,131, 96,872)
School costs^c^	4789^b^	2888^b^	1901^b^	3472 (1958, 4987)	2724 (1573, 3875)
Total public sector costs^c^	5984 (4812, 7623)	3203 (3129, 3291)	2781 (1596, 4447)	2969 (1525, 4414)	4101 (2791, 5411)
Total societal costs^d^	6536 (5142, 8647)	3275 (3188, 3392)	3262 (1853, 5317)	3216 (1674, 4758)	4667 (3199, 6135)
Total societal costs^e^	9389 (7389, 12,909)	3278 (3179, 3409)	6111 (4074, 9608)	4772 (3166, 6378)	5237 (3965, 6509)

*Note*: Note that school costs and total societal costs include costs of mainstream education, special schools and special units attached to mainstream schools but excludes provision of special education services in mainstream schools.

^a^ Bias‐corrected bootstrap confidence interval; 95% CI refers to 95% confidence interval.

^b^ Not possible to estimate a confidence interval.

^c^ Includes school costs (mainstream, special and special units attached to mainstream schools) but excludes provision of additional educational support in mainstream schools.

^d^ Includes public sector (healthcare and social care costs and education costs excluding provision of additional educational support in mainstream schools), productivity and additional care costs.

^e^ Includes public sector (healthcare and social care costs and education costs including provision of additional educational support in mainstream schools), productivity and additional care costs based on a reduced sample of 158 children (76 in the extremely preterm group and 82 in the term group).

The cost of SEN provision in mainstream schools was excluded from the main cost comparisons reported above due to a higher degree of missing information for categories of education services captured on the teacher completed questionnaires. Including these costs in the societal cost calculations based on a reduced sample of 158 children (76 extremely preterm children and 82 classmates born at term) who had complete data across all categories of service utilisation increased the adjusted cost difference to £4772 (95% CI £3166, £6378) (Table 2). Including costs associated with SEN provision in the societal cost calculations using multiple imputations to account for missing data increased the adjusted mean total societal cost difference to £5254 (95% CI £3979, £6530) (Table S3).

### 3.3 Health utility values

The proportion reporting suboptimal levels of function was higher in the extremely preterm group compared with classmates across HUI2 and HUI3 attributes (Table 3). When the analysis was stratified by gestational age, there were higher proportions of the suboptimal level of function for the extremely preterm children compared with classmates (Table 3).

**TABLE 3:** Number (%) of children with suboptimal levels of function (below level 1) within attributes of HUI2 and HUI3

Attribute	Gestational age at birth				All extremely preterm(*n* = 161)	Classmates (*n* = 120)
	23 weeks (*n* = 11)	24 weeks (*n* = 21)	25 weeks (*n* = 57)	26 weeks (*n* = 72)		
*HUI2*						
Sensation	9 (81.8)	16 (76.2)	32 (56.1)	31 (43.1)	88 (54.7)	22 (18.6)
Mobility	3 (27.3)	1 (4.8)	9 (15.8)	6 (8.3)	19 (11.8)	0
Emotion	4 (36.4)	8 (38.1)	27 (47.4)	34 (47.2)	73 (45.3)	26 (22)
Cognition	7 (63.6)	15 (71.4)	32 (56.1)	40 (55.6)	94 (58.4)	16 (13.6)
Self‐Care	6 (54.5)	2 (9.5)	9 (15.8)	16 (22.2)	33 (20.5)	1 (0.8)
Pain	3 (27.3)	2 (9.5)	15 (26.3)	23 (31.9)	43 (26.7)	20 (16.9)
*HUI3*						
Vision	6 (54.5)	7 (33.3)	21 (36.8)	22 (30.6)	56 (34.8)	21 (17.8)
Hearing	2 (18.2)	4 (19)	6 (10.5)	6 (8.3)	18 (11.2)	2 (1.7)
Speech	5 (45.5)	11 (52.4)	18 (31.6)	13 (18.1)	47 (29.2)	2 (1.7)
Emotion	3 (27.3)	3 (14.3)	15 (26.3)	19 (26.4)	40 (24.8)	13 (11)
Pain	3 (27.3)	4 (19)	16 (28.1)	24 (33.3)	47 (29.2)	16 (13.6)
Ambulation	3 (27.3)	1 (4.8)	9 (15.8)	6 (8.3)	19 (11.8)	0
Dexterity	6 (54.5)	3 (14.3)	10 (17.5)	9 (12.5)	28 (17.4)	0
Cognition	7 (63.6)	15 (71.4)	32 (56.1)	40 (55.6)	94 (58.4)	16 (13.6)

The extremely preterm group had lower mean scores on both HUI instruments and associated algorithms than their classmates born at term (Table 4). The adjusted mean utility decrement ranged from 0.13 (95% CI 0.09, 0.18) for scores generated via the UK multiattribute utility function value set for the HUI2 to 0.28 (95% CI 0.18, 0.37) for health utilities generated via the Canadian value set for the HUI3. When the analyses were stratified by gestational age, the mean utility decrement was greatest for the 23‐week gestation group and smallest for those born at 26 weeks' gestation when compared with children born at term (Tables S4, S5, S6, S7 and S8). The adjusted mean utility decrement for the 23‐week group compared with classmates ranged from 0.19 (95% CI 0.04, 0.34) for the HUI2 UK statistical inference value set to 0.48 (95% CI 0.01, 0.96) for HUI3 Canadian value set. The adjusted mean utility decrement for the 26‐week group ranged from 0.13 (95% CI 0.08, 0.18) for the HUI2 UK statistical inference value set to 0.23 (95% CI 0.13, 0.34) for HUI3 Canadian value set (Table S4).

**TABLE 4:** HUI multiattribute utility scores by HUI measure and utility algorithm

Outcome	Extremely preterm		Classmates		Extremely preterm vs. classmates		
	*N*	Mean (SD) utility	*N*	Mean (SD) utility	Unadjusted difference (95% CI), complete cases	Adjusted difference (95% CI), complete cases	Adjusted difference (95% CI), multiple imputation
HUI2 UK MAUF	162	0.770 (0.187)	118	0.930 (0.088)	−0.160 (−0.200, −0.126)	−0.15 (−0.02, −0.11)	−0.16 (−0.19, −0.12)
HUI2 UK SI	162	0.789 (0.176)	118	0.930 (0.089)	−0.142 (−0.177, −0.108)	−0.13 (−0.18, −0.09)	−0.14 (−0.17, −0.10)
HUI2 Canada MAUF	162	0.810 (0.185)	118	0.956 (0.061)	−0.146 (−0.180, −0.115)	−0.14 (−0.19, −0.10)	−0.14 (−0.18, −0.11)
HUI3 Canada	161	0.685 (0.324)	120	0.947 (0.118)	−0.262 (−0.339, −0.194)	−0.28 (−0.37, −0.18)	−0.27 (−0.35, −0.19)

*Note*: HUI2 Canada MAUF = HUI2 utility score generated via the Canadian multiattribute utility function value set for Canada.

HUI3 Canada MAUF = HUI3 utility score generated via the Canadian multiattribute utility function value set for Canada.

HUI2 UK MAUF = HUI2 utility score generated via the UK multiattribute utility function value set.

HUI2 UK MAUF = HUI2 utility score generated via the UK statistical inference value set.

## 4 COMMENT

### 4.1 Principal findings

We report here the impact of extremely preterm birth (≤26 completed weeks of gestation) on economic costs and health‐related quality of life at 11‐years of age using a nationally representative sample of extremely preterm children born in England (EPICure2 cohort) and a comparison group of classmates born at term. Consistent with the findings from our previous analyses of the 1995 EPICure cohort,^38^ we found evidence that extremely preterm birth increased utilisation of health and social care services and special educational needs provision in late childhood. Costs to families in the form of out‐of‐pocket medical expenses and the informal care and broader costs to society derived from the valuation of work absence were also elevated in the extremely preterm group. Extremely preterm birth was also associated with lower utility scores compared with children born at term.

### 4.2 Strengths of the study

Our analysis was based on a prospective population‐based sample drawn from defined geographic areas of England; hence selection biases are unlikely to represent a major concern. We extended our cost estimates to include direct non‐medical costs borne by families and indirect costs associated with lost productivity. The 2006 EPICure data thus provided a more complete picture of the cost of extremely preterm birth to society than the 1995 data, which were restricted to public sector costs only. Our estimates of the utility decrements for the HUI2 and HUI3 can be used to inform cost‐utility analyses as they are based on values derived from Canadian and UK general populations, making them applicable for use across a wide range of evaluative studies and settings.

### 4.3 Limitations of the data

The term‐born controls in our study were sampled from the classmates of preterm‐born children in mainstream schools only. Recruiting a term‐born classmate for every preterm child in special school would result in a substantially higher proportion of controls with complex special educational needs relative to the general population. We acknowledge that this sampling approach may have resulted in the recruitment of a term‐born control group that is slightly healthier than the general population and therefore we may have slightly under‐estimated the costs for term‐born children. In addition, the response rate was lower for the teacher completed questionnaires. This meant that our data did not fully capture the costs of SEN provision due to poor questionnaire completion rates. In the EPICure1 cohort, 61% of the extremely preterm children accessed SEN services across educational settings at age 11 compared with 14% of children born at term.^39^ This would suggest our cost estimates are likely to represent the lower bound of costs and burden associated with extremely preterm birth to the public sector and to society more broadly.

Teachers completing questionnaires were not blinded to gestational age and may have been aware of the birth history of the children being assessed as this was required to be communicated in order for schools to identify potential controls. The study questionnaire was proxy completed by parents and teachers of study participants, reflecting their perspectives rather than that of the children, including the health status assessment. Other studies have revealed discrepancies in the descriptions of children's health‐related quality of life, as measured using the HUI measures, provided by preterm children and their parents.^40^, ^41^ Furthermore, the underpinning preference‐based values (or utilities) attached to health states within the HUI health status classification systems were derived from surveys of adults, necessitated by the absence of national value sets for the HUI classification systems derived from childhood or adolescent samples. The development of preference‐based value sets for health‐related quality of life measures such as the HUI2 and HUI3 has largely overlooked the normative question of whose values are most valid for informing clinical and resource allocation decisions in the paediatric context. Arguably, the application of childhood or adolescent‐derived values could have led to a different pattern of results.

### 4.4 Interpretation

The present analyses included direct non‐medical costs borne by families and indirect costs associated with lost productivity and so are not directly comparable to our previous cost estimates based on the 1995 EPICure cohort,^38^ which were limited to public sector costs. Furthermore, the extremely preterm group in the 1995 EPICure cohort was limited to children born at ≤25 weeks' gestation. In an additional analysis aimed at comparing our results with those based on the 1995 EPICure cohort, we restricted our cost estimates to children born at ≤25 weeks' gestation and our study perspective to encompass public sector costs only. This generated mean unadjusted and adjusted public sector cost differences of £3037 (95% CI £1492, £5386) and £2376 (95% CI £1138, £3614) between the extremely preterm group and their term‐born classmates. The analogous estimates from the 1995 cohort were £3115 (95% CI £2018, £4225) and £2022 (95% CI £862, £3181), respectively, when valued at comparable 2019 prices. This suggests a sustained burden on public sector services in the late childhood years for extremely preterm birth despite improvements in neonatal care in the 11 years between the 1995 and 2006 EPICure cohorts.

In the decade long period that separates the 1995 (EPICure1) and 2006 (EPICure2) birth cohorts, there were notable improvements in the perinatal care of mothers and newborn infants with substantial improvement in survival after preterm birth.^17^, ^18^ Restricting the EPICure2 data to children born at ≤25 weeks' gestation generated a mean adjusted HUI3 multiattribute utility decrement associated with extremely preterm birth of 0.31 (95% CI 0.16, 0.45). The corresponding mean adjusted HUI3 multiattribute utility decrement estimated for the EPICure1 cohort was 0.28 (95% CI 0.20, 0.36).^38^ Our data would thus suggest that advances in neonatal care from 1995 to 2006 have not translated into health‐related quality of life improvements in the late childhood years for extremely preterm children.

The greatest differences in functional outcomes within the HUI measures applied were observed in the sensation, cognition and emotion attributes of the HUI2, and vision and cognition attributes of the HUI3. However, the multiattribute utility scores generated from the HUI2 and HU3 instruments are not directly comparable because of differences in the population samples and characteristics of the Canadian and UK populations surveyed and the valuation protocols applied. The utility decrement associated with extremely preterm birth was greatest for the HUI3 (mean utility decrement 0.28) based on a multiattribute utility function derived from a survey of Canadian adults and smallest for the HUI2 with an estimated mean decrement of 0.13 derived from a statistical inference model based on a survey of UK adult preferences.^33^ These findings are consistent with a mean utility decrement of 0.13 reported by Zwicker and Harris 2007^42^ for the HUI2 and 0.28 for the HUI3 based on 11‐year‐olds surveyed in the EPICure1 cohort.^38^ The discrepancy between our estimates of utility decrement based on the HUI2 and HUI3 is a consequence of differences in the populations surveyed to generate the respective HUI2 and HUI3 value sets and the valuation protocols applied.

## 5 CONCLUSIONS

In conclusion, despite improvements in neonatal care in the decade‐long period that separate the 1995 and 2006 EPICure cohorts, there is no evidence that the adverse economic impact of extremely preterm birth in the late childhood years has ameliorated. Further longitudinal studies conducted from multiple perspectives are needed to understand the magnitude, trajectory and underpinning mechanisms of economic outcomes following extremely preterm birth.

## AUTHOR'S CONTRIBUTION

NM, SJ, SP and DW conceived the original study design. NM and SJ were joint project leads. SP lead design of the health economics component of the project. YN coded the data and put together the data sets for analysis. FA and KK conducted costing exercise, analyse the data and drafted wrote the first draft of the manuscript. All authors participated in drafting, proof‐reading and approval of the final manuscript.

## CONFLICT OF INTEREST

SP receives support as a NIHR Senior Investigator (NF‐SI‐0616‐10103) and from the NIHR Applied Research Collaboration Oxford and Thames Valley. The views contained within this paper are those of the authors and not necessarily of the funders. NM declares Consulting fees with Novartis outside the published work and receives part funding from the Department of Health’s National Institute for Health Research Biomedical Research Centre’s funding scheme at UCLH/UCL. All other authors have no conflict of interest to report.

## Supporting information


Table S1

Table S2

Table S3

Table S4

Table S5

Table S6

Table S7

Table S8

Appendix S1

## Data Availability

Data subject to third party restrictions
