# Supplementary material for: Economic costs and health utility values associated with extremely preterm birth: Evidence from the EPICure2 cohort study
Source: Paediatr Perinat Epidemiol. 2022 Jul 13;36(5):696–705. doi: 10.1111/ppe.12906 (PMC9543967; doi:10.1111/ppe.12906)
Supplement: Supplementary file 1 — Table S1 [file PPE-36-696-s003.docx]

eTable 1: Resource use and unit costs of resource items (UK pound sterling, 2019). Resource use values are given as number with complete data (n) and the mean and standard deviation (SD) for each item of resource use unless otherwise stated.

|  | **Extremely preterm children (N = 200)** | |  | **Classmates (N = 143)** | |  |  |
| --- | --- | --- | --- | --- | --- | --- | --- |
| **Resource use variable** | **n** | **Mean (SD) resource use** |  | **n** | **Mean (SD) resource** | **Unit cost or cost range** | **Sources of unit costs** |
| **Medications (items)** |  |  |  |  |  |  |  |
| Medication for respiratory /chest problems | 172 | 0.791 (2.177) |  | 125 | 0.2 (0.635) | 1.31-1505.818 | ^25,26,28^ |
| Medication to control fits | 172 | 0.145 (0.842) |  | 125 | 0.04 (0.447) | 66.39-211.19 | ^22^ |
| Other medication | 172 | 1.186 (1.832) |  | 125 | 0.224 (0.923) | 0.056-38817.75 | ^22,27^ |
| Community health and social care (contacts) |  |  |  |  |  |  |  |
| General Practitioner, contacts | 161 | 1.547 (1.972) |  | 119 | 0.908 (1.142) | 39 | ^23^ |
| Practice Nurse, contacts | 169 | 0.426 (0.998) |  | 121 | 0.132 (0.515) | 10.85 | ^23^ |
| Community Nurse, contacts | 169 | 0.189 (0.866) |  | 123 | 0.024 (0.271) | 15.333 | ^23^ |
| Community Paediatrician, contacts | 166 | 0.277 (0.693) |  | 123 | 0.016 (0.18) | 36.333 | ^23^ |
| Dentist, contacts | 158 | 1.785 (1.425) |  | 116 | 1.569 (0.815) | 44.333 | ^23^ |
| Orthodontist, contacts | 167 | 0.503 (1.326) |  | 122 | 0.246 (0.796) | 15 | ^23^ |
| Optician, contacts | 163 | 0.896 (0.872) |  | 119 | 0.689 (0.81) | 7.5 | ^23^ |
| Chiropodist, contacts | 169 | 0.03 (0.23) |  | 123 | 0.171 (1.894) | 15 | ^23^ |
| Physiotherapist, contacts | 165 | 1.121 (5.503) |  | 123 | 0.024 (0.271) | 30 | ^23^ |
| Speech therapist, contacts | 166 | 3.084 (12.677) |  | 123 | 0.016 (0.18) | 30 | ^23^ |
| Audiologist | 166 | 0.319 (0.991) |  | 123 | 0.033 (0.178) | 15 | ^23^ |
| Social Worker | 166 | 0.229 (1.013) |  | 123 | 0.073 (0.726) | 34 | ^23^ |
| Home Visitor / Volunteer | 168 | 0.964 (6.908) |  | 123 | 0 (0) | 24.75 | ^23^ |
| Counsellor | 169 | 0.633 (4.446) |  | 123 | 0.48 (4.726) | 30 | ^23^ |
| Clinical psychologist | 168 | 0.262 (1.368) |  | 123 | 0.114 (1.095) | 30 | ^23^ |
| Psychiatrist | 168 | 0.006 (0.077) |  | 123 | 0 (0) | 30 | ^23^ |
| Osteopath | 169 | 0.284 (1.943) |  | 123 | 0.057 (0.484) | 30 | ^23^ |
| Home Teacher / Tutor (portage) | 168 | 0.119 (1.543) |  | 123 | 0 (0) | 45 | ^23^ |
| Home Teacher / Tutor (other) | 169 | 0.621 (5.15) |  | 123 | 0 (0) | 45 | ^23^ |
| Orthoptists | 168 | 0.143 (0.668) |  | 122 | 0 (0) | 15 | ^23^ |
| Orthotist | 169 | 0.195 (0.726) |  | 123 | 0 (0) | 15 | ^23^ |
| Occupational therapist | 135 | 0.696 (5.015) |  | 117 | 0 (0) | 30 | ^23^ |
| Other (specify) | 186 | 0.28 (1.069) |  | 138 | 0.116 (0.663) | 15-72.667 | ^23^ |
| **Secondary care** |  |  |  |  |  |  |  |
| Accident & Emergency, hours | 172 | 0.471 (0.625) |  | 125 | 0.216 (0.468) | 160-640 | ^21^ |
| Hospital Day Unit, hours | 172 | 0.453 (0.605) |  | 125 | 0.184 (0.389) | 420-1680 | ^21^ |
| Hospital Outpatients, hours | 172 | 1.192 (2.558) |  | 125 | 0.28 (0.799) | 125-2125 | ^21^ |
| Admitted for breathing difficulties, days | 172 | 0.006 (0.076) |  | 125 | 0.016 (0.126) |  | ^21^ |
| Admitted for surgery, days | 172 | 0.081 (0.452) |  | 125 | 0.048 (0.307) | 507-7862 | ^21^ |
| Admitted to intensive care unit (ICU), days | 172 | 0.006 (0.076) |  | 125 | 0 (0) | 559 | ^21^ |
| Admitted for other reasons, days | 172 | 0.035 (0.263) |  | 125 | 0.032 (0.358) | 4161.2 | ^21^ |
| Any other long-term illness, days | 172 | 0 (0) |  | 125 | 0.008 (0.089) |  | ^21^ |
| **Additional costs to families** |  |  |  |  |  |  |  |
| Food, amount in £ | 168 | 19.565 (155.88) |  | 123 | 0 (0) | 3-1800 | Parent reported |
| Bedding, amount in £ | 169 | 9.024 (107.75) |  | 123 | 0 (0) | 3-1400 | Parent reported |
| Washing/Laundry, amount in £ | 164 | 0.951 (4.476) |  | 123 | 0 (0) | 3-30.0 | Parent reported |
| Clothing, amount in £ | 164 | 10.573 (110.051) |  | 123 | 0 (0) | 20-1400 | Parent reported |
| Cost of visiting hospital or GP, amount in £ | 165 | 6.57 (45.85) |  | 122 | 0.328 (3.621) | 6-490 | Parent reported |
| Child Care, amount in £ | 167 | 7.305 (83.863) |  | 123 | 0 (0) | 60-1080 | Parent reported |
| Help with housework, amount in £ | 169 | 11.302 (109.205) |  | 123 | 0 (0) | 30-1400 | Parent reported |
| Telephone bills, amount in £ | 170 | 0.141 (1.841) |  | 123 | 0 (0) | 24 | Parent reported |
| Other additional costs, amount in £ | 170 | 28.076 (205.586) |  | 123 | 4.065 (45.083) | 5-2000 | Parent reported |
| Therapies for your child, amount in £ | 168 | 21.31 (106.362) |  | 123 | 1.626 (18.033) | 15-980 | Parent reported |
| Repairs or adaptations to your home, amount in £ | 167 | 0 (0) |  | 123 | 0 (0) |  | Parent reported |
| Special equipment for your child, amount in £ | 169 | 31.278 (231.475) |  | 123 | 0 (0) | 05-2795 | Parent reported |
| Other expenses, amount in £ | 164 | 28.94 (351.117) |  | 122 | 0 (0) | 50-4496.24 | Parent reported |
| **Time off work** |  |  |  |  |  |  |  |
| Parent, days | 171 | 2.994 (19.829) |  | 125 | 0.368 (1.133) | 111.72 | ^32^ |
| Partner, days | 170 | 0.424 (1.638) |  | 123 | 0.163 (0.751) | 111.72-121.64 | ^32^ |
| Relative/friend, days | 170 | 0.076 (0.499) |  | 125 | 0.008 (0.089) | 111.72 | ^32^ |
| **Education services** |  |  |  |  |  |  |  |
| Mainstream school, item | 200 | 0.865 (0.343) |  | 143 | 0.993 (0.084) | 2888.25 | ^31^ |
| Mainstream school with special unit attached item | 200 | 0.015 (0.122) |  | 143 | 0 (0) | 25794.07 | ^31^ |
| Special school, item | 200 | 0.11 (0.314) |  | 143 | 0 (0) | 25794.07 | ^31^ |
| Other, item | 200 | 0.01 (0.1) |  | 143 | 0.007 (0.084) |  | ^31^ |
| Child has an EHC plan, item | 150 | 0.32 (0.468) |  | 117 | 0 (0) | 153.846 | ^31^ |
| Individual education/behaviour plan, hours per week | 118 | 2.877 (8.8) |  | 115 | 0 (0) | 5.13 | ^31^ |
| One-to-one special needs provision, hours per week | 137 | 3.686 (9.431) |  | 116 | 0.009 (0.093) | 5.13 | ^31^ |
| Small group special needs provision, hours per week | 123 | 4.5 (10.047) |  | 117 | 0.068 (0.653) | 5.13 | ^31^ |
| Outreach teacher(s), hours per week | 139 | 0.038 (0.215) |  | 117 | 0 (0) | 45 | ^31^ |
| Educational psychologist, hours per week | 130 | 0.009 (0.088) |  | 116 | 0 (0) | 45 | ^31^ |
