# Supplementary material for: Economic costs and health utility values associated with extremely preterm birth: Evidence from the EPICure2 cohort study
Source: Paediatr Perinat Epidemiol. 2022 Jul 13;36(5):696–705. doi: 10.1111/ppe.12906 (PMC9543967; doi:10.1111/ppe.12906)
Supplement: Supplementary file 2 — Table S2 [file PPE-36-696-s004.docx]

eTable 2: Predictors of societal costs excluding provision of special educational support services in mainstream schools at age 11 in UK pound sterling and 2019 prices, complete case analysis

|  | **Model 1** | | |  | **Model 2** | | |
| --- | --- | --- | --- | --- | --- | --- | --- |
| Variable | Coef (SE)^a^ | Cost ratio (95% CI)^b^ | Cost difference (95% CI)^b^, £ |  | Coef (SE)^a^ | Cost ratio (95% CI)^b^ | Cost difference (95% CI)^b^, £ |
| Gestational age at birth |  |  |  |  |  |  |  |
| 23 weeks |  |  |  |  | 1.0 (0.45) | 2.71 (1.11, 6.6) | 5678 (-2106, 13463) |
| 24 weeks |  |  |  |  | 0.75 (0.26) | 2.12 (1.28, 3.53) | 3732 (294, 7170) |
| 25 weeks |  |  |  |  | 0.43 (0.18) | 1.54 (1.08, 2.2) | 1794 (104, 3483) |
| 26 weeks |  |  |  |  | 0.68 (0.17) | 1.98 (1.42, 2.77) | 3268 (1340, 5196) |
| All extremely preterm | 0.63 (0.13) | 1.87 (1.44, 2.43) | 2916 (1609, 4224) |  | - | - | - |
| Age (years) | 0.05 (0.12) | 1.06 (0.84, 1.34) | 275 (-914, 1464) |  | 0.06 (0.12) | 1.06 (0.83, 1.35) | 288 (-949, 1524) |
| IMD ≤5^c^ | -0.1 (0.14) | 0.90 (0.69, 1.18) | -522 (-1887, 843) |  | -0.06 (0.14) | 0.94 (0.71, 1.25) | -305 (-1729, 1120) |
| Male | -0.37 (0.13) | 0.69 (0.54, 0.88) | -1843 (-3123, -562) |  | -0.35 (0.13) | 0.7 (0.54, 0.91) | -1728 (-3041, -415) |
| Non-white | -0.38 (0.16) | 0.69 (0.5, 0.95) | -1728 (-3113, -344) |  | -0.38 (0.17) | 0.69 (0.49, 0.96) | -1740 (-3175, -304) |
| Smoker in the house | -0.06 (0.16) | 0.94 (0.68, 1.29) | -311 (-1829, 1208) |  | -0.08 (0.17) | 0.93 (0.67, 1.29) | -362 (-1902, 1179) |
| Constant | 8.43 (0.13) | 4568 (3524, 5921)^d^ | - |  | 8.39 (0.14) | 4418 (3369, 57924)^d^ | - |
| ^a^Cofficient (Standard error)  ^b^95% confidence intervals  ^c^Index of multiple deprivation  ^d^Exponential of the coefficient for the regression intercept | | | | | | | |
