# Supplementary material for: Economic costs and health utility values associated with extremely preterm birth: Evidence from the EPICure2 cohort study
Source: Paediatr Perinat Epidemiol. 2022 Jul 13;36(5):696–705. doi: 10.1111/ppe.12906 (PMC9543967; doi:10.1111/ppe.12906)
Supplement: Supplementary file 3 — Table S3 [file PPE-36-696-s007.docx]

Table 3: HUI multi-attribute utility scores stratified by comparator group and gestational age

|  | Extremely preterm | |  | Term-born classmates | |  | Extremely preterm versus term born classmate | | |
| --- | --- | --- | --- | --- | --- | --- | --- | --- | --- |
|  |  | |  |  | |  |  | | |
| Outcome | N | Mean (SD) utility |  | N | Mean (SD) utility |  | Unadjusted difference (95% CI), complete cases | Adjusted difference (95% CI), complete cases | Adjusted difference (95% CI), multiple imputation |
| *≤23 weeks gestation* |  |  |  |  |  |  |  |  |  |
| HUI2 (UK MAUF) | 11 | 0.683 (0.058) |  | 118 | 0.93 (0.008) |  | -0.247 (-0.369, -0.13) | -0.19 (-0.38, 0.01) | -0.22 (-0.38, -0.06) |
| HUI2 (UK SI) | 11 | 0.708 (0.052) |  | 118 | 0.93 (0.008) |  | -0.222 (-0.32, -0.119) | -0.17 (-0.35, 0.02) | -0.19 (-0.34, -0.04) |
| HUI2 (Canada MAUF) | 11 | 0.707 (0.061) |  | 118 | 0.956 (0.006) |  | -0.249 (-0.391, -0.146) | -0.2 (-0.41, 0.01) | -0.22 (-0.39, -0.05) |
| HUI3 (Canada MAUF) | 11 | 0.444 (0.118) |  | 120 | 0.947 (0.011) |  | -0.502 (-0.75, -0.284) | -0.49 (-1.00, 0.12) | -0.48 (-0.96, -0.01) |
| *24^+0^–24^+6^ weeks* |  |  |  |  |  |  |  |  |  |
| HUI2 (UK MAUF) | 21 | 0.759 (0.037) |  | 118 | 0.93 (0.008) |  | -0.171 (-0.262, -0.108) | -0.18 (-0.3, -0.06) | -0.17 (-0.26, -0.07) |
| HUI2 (UK SI) | 21 | 0.795 (0.035) |  | 118 | 0.93 (0.008) |  | -0.136 (-0.222, -0.081) | -0.14 (-0.25, -0.03) | -0.13 (-0.22, -0.05) |
| HUI2 (Canada MAUF) | 21 | 0.805 (0.037) |  | 118 | 0.956 (0.006) |  | -0.151 (-0.241, -0.094) | -0.16 (-0.27, -0.05) | -0.15 (-0.23, -0.06) |
| HUI3 (Canada MAUF) | 21 | 0.672 (0.062) |  | 120 | 0.947 (0.011) |  | -0.274 (-0.418, -0.17) | -0.32 (-0.6, -0.05) | -0.28 (-0.5, -0.07) |
| *25^+0^–25^+6^ weeks* |  |  |  |  |  |  |  |  |  |
| HUI2 (UK MAUF) | 57 | 0.771 (0.025) |  | 118 | 0.93 (0.008) |  | -0.159 (-0.213, -0.107) | -0.15 (-0.22, -0.08) | -0.16 (-0.21, -0.1) |
| HUI2 (UK SI) | 57 | 0.787 (0.026) |  | 118 | 0.93 (0.008) |  | -0.143 (-0.200, -0.094) | -0.13 (-0.2, -0.07) | -0.14 (-0.19, -0.08) |
| HUI2 (Canada MAUF) | 57 | 0.814 (0.025) |  | 118 | 0.956 (0.006) |  | -0.142 (-0.201, -0.097) | -0.14 (-0.2, -0.07) | -0.14 (-0.19, -0.09) |
| HUI3 (Canada MAUF) | 57 | 0.675 (0.046) |  | 120 | 0.947 (0.011) |  | -0.272 (-0.375, -0.189) | -0.26 (-0.4, -0.11) | -0.27 (-0.4, -0.14) |
| *26^+0^–26^+6^ weeks* |  |  |  |  |  |  |  |  |  |
| HUI2 (UK MAUF) | 73 | 0.785 (0.02) |  | 118 | 0.93 (0.008) |  | -0.145 (-0.191, -0.106) | -0.14 (-0.21, -0.08) | -0.15 (-0.2, -0.1) |
| HUI2 (UK SI) | 73 | 0.8 (0.019) |  | 118 | 0.93 (0.008) |  | -0.130 (-0.17, -0.09) | -0.13 (-0.19, -0.07) | -0.13 (-0.18, -0.08) |
| HUI2 (Canada MAUF) | 73 | 0.823 (0.022) |  | 118 | 0.956 (0.006) |  | -0.133 (-0.181, -0.091) | -0.14 (-0.2, -0.08) | -0.13 (-0.18, -0.09) |
| HUI3 (Canada MAUF) | 72 | 0.733 (0.034) |  | 120 | 0.947 (0.011) |  | -0.214 (-0.291, -0.151) | -0.25 (-0.38, -0.11) | -0.23 (-0.34, -0.13) |
| HUI2 Canada MAUF = HUI2 utility score generated via the Canadian multi-attribute utility function value set for Canada  HUI3 Canada MAUF = HUI3 utility score generated via the Canadian multi-attribute utility function value set for Canada  HUI2 UK MAUF = HUI2 utility score generated via the UK multi-attribute utility function value set  HUI2 UK MAUF = HUI2 utility score generated via the UK statistical inference value set | | | | | | | | | |
