# Supplementary material for: Economic costs and health utility values associated with extremely preterm birth: Evidence from the EPICure2 cohort study
Source: Paediatr Perinat Epidemiol. 2022 Jul 13;36(5):696–705. doi: 10.1111/ppe.12906 (PMC9543967; doi:10.1111/ppe.12906)
Supplement: Supplementary file 4 — Table S4 [file PPE-36-696-s005.docx]

eTable 4: Predictors of HUI2 (UK statistical inference model) utility scores during the 11th year of life

| Variable | **Model 1** | | |  | **Model 2** | | |
| --- | --- | --- | --- | --- | --- | --- | --- |
| Gestational age at birth | Coef (SE)^a^ | Utility ratio (95% CI)^b^ | Utility difference (95% CI)^b^ |  | Coef (SE)^a^ | Utility ratio (95% CI)^b^ | Utility difference (95% CI)^b^ |
| 23 weeks |  |  |  |  | -1.13 (0.4) | 0.32 (0.15, 0.71) | -0.17 (-0.29, -0.05) |
| 24 weeks |  |  |  |  | -1.02 (0.27) | 0.36 (0.21, 0.61) | -0.14 (-0.24, -0.05) |
| 25 weeks |  |  |  |  | -0.98 (0.19) | 0.38 (0.26, 0.55) | -0.13 (-0.19, -0.07) |
| 26 weeks |  |  |  |  | -0.96 (0.19) | 0.38 (0.26, 0.55) | -0.13 (-0.18, -0.08) |
| All extremely preterm | -0.99 (0.15) | 0.37 (0.28, 0.5) | -0.13 (-0.18, -0.09) |  | - | - | - |
| Age (years) | -0.1 (0.13) | 0.9 (0.7, 1.16) | -0.02 (-0.06, 0.02) |  | -0.1 (0.13) | 0.9 (0.7, 1.16) | -0.02 (-0.06, 0.03) |
| IMD ≤5^c^ | 0.03 (0.15) | 1.03 (0.76, 1.39) | 0 (-0.04, 0.05) |  | 0.03 (0.16) | 1.03 (0.76, 1.4) | 0 (-0.04, 0.05) |
| Male | 0.16 (0.14) | 1.18 (0.89, 1.56) | 0.03 (-0.02, 0.07) |  | 0.15 (0.15) | 1.17 (0.87, 1.56) | 0.02 (-0.02, 0.07) |
| Non-white | -0.22 (0.17) | 0.8 (0.57, 1.13) | -0.04 (-0.1, 0.02) |  | -0.22 (0.18) | 0.81 (0.57, 1.14) | -0.04 (-0.11, 0.03) |
| Smoker in the house | -0.06 (0.19) | 0.94 (0.65, 1.37) | -0.01 (-0.07, 0.05) |  | -0.05 (0.2) | 0.95 (0.65, 1.4) | -0.01 (-0.09, 0.07) |
| Constant | 2.51 (0.16) | 0.92 (0.89, 0.94)^d^ | - |  | 2.51 (0.16) | 0.92 (0.89, 0.94)^d^ | - |
| ^a^Cofficient (Standard error)  ^b^95% confidence intervals  ^b^Index of multiple deprivation  ^d^Exponential of the coefficient for the regression intercept | | | | | | | |
