# Supplementary material for: Economic costs and health utility values associated with extremely preterm birth: Evidence from the EPICure2 cohort study
Source: Paediatr Perinat Epidemiol. 2022 Jul 13;36(5):696–705. doi: 10.1111/ppe.12906 (PMC9543967; doi:10.1111/ppe.12906)
Supplement: Supplementary file 5 — Table S5 [file PPE-36-696-s008.docx]

eTable 5: Predictors of HUI2 (UK MAUF model) utility score at 11^th^ year of life

|  | **Model 2** | | |  | **Model 2** | | |
| --- | --- | --- | --- | --- | --- | --- | --- |
| Variable | Coef (SE)^a^ | Utility ratio (95% CI)^b^ | Utility difference (95% CI)^b^ |  | Coef (SE)^a^ | Utility ratio (95% CI)^b^ | Utility difference (95% CI)^b^ |
| Gestational age at birth |  |  |  |  |  |  |  |
| 23 weeks |  |  |  |  | -1.19 (0.39) | 0.3 (0.14, 0.65) | -0.19 (-0.33, -0.05) |
| 24 weeks |  |  |  |  | -1.16 (0.26) | 0.31 (0.19, 0.53) | -0.18 (-0.29, -0.07) |
| 25 weeks |  |  |  |  | -1.05 (0.19) | 0.35 (0.24, 0.51) | -0.15 (-0.21, -0.09) |
| 26 weeks |  |  |  |  | -1.01 (0.18) | 0.36 (0.25, 0.52) | -0.14 (-0.21, -0.08) |
| All extremely preterm | -1.06 (0.15) | 0.35 (0.26, 0.46) | -0.15 (-0.2, -0.11) |  |  |  |  |
| Age (years) | -0.09 (0.12) | 0.92 (0.72, 1.17) | -0.02 (-0.06, 0.03) |  | -0.09 (0.13) | 0.91 (0.71, 1.17) | -0.02 (-0.06, 0.03) |
| IMD ≤5^c^ | 0 (0.15) | 1 (0.75, 1.34) | 0 (-0.05, 0.05) |  | 0.01 (0.15) | 1.01 (0.75, 1.36) | 0 (-0.06, 0.06) |
| Male | 0.13 (0.14) | 1.14 (0.87, 1.5) | 0.02 (-0.02, 0.07) |  | 0.13 (0.14) | 1.14 (0.86, 1.51) | 0.02 (-0.03, 0.07) |
| Non-white | -0.15 (0.17) | 0.86 (0.62, 1.2) | -0.03 (-0.09, 0.04) |  | -0.15 (0.17) | 0.86 (0.62, 1.21) | -0.03 (-0.1, 0.05) |
| Smoker in the house | -0.12 (0.19) | 0.89 (0.62, 1.28) | -0.02 (-0.09, 0.05) |  | -0.11 (0.19) | 0.89 (0.61, 1.3) | -0.02 (-0.11, 0.07) |
| Constant | 2.5 (0.15) | 0.92 (0.89, 0.94)^d^ |  |  | 2.5 (0.16) | 0.92 (0.89, 0.94)^d^ |  |
| ^a^Cofficient (Standard error)  ^b^95% confidence intervals  ^b^Index of multiple deprivation  ^d^Exponential of the coefficient for the regression intercept | | | | | | | |
