# Supplementary material for: Economic costs and health utility values associated with extremely preterm birth: Evidence from the EPICure2 cohort study
Source: Paediatr Perinat Epidemiol. 2022 Jul 13;36(5):696–705. doi: 10.1111/ppe.12906 (PMC9543967; doi:10.1111/ppe.12906)
Supplement: Supplementary file 6 — Table S6 [file PPE-36-696-s002.docx]

eTable 6: Predictors HUI2 (Canada MAUF model) utility score during the 11^th^ year of life

|  | **Model 1** | | |  | **Model 2** | | |
| --- | --- | --- | --- | --- | --- | --- | --- |
| Variable | Coef (SE)^a^ | Utility ratio (95% CI)^b^ | Utility difference (95% CI)^b^ |  | Coef (SE)^a^ | Utility ratio (95% CI)^b^ | Utility difference (95% CI)^b^ |
| Gestational age at birth |  |  |  |  |  |  |  |
| 23 weeks |  |  |  |  | -1.57 (0.44) | 0.21 (0.09, 0.5) | -0.2 (-0.37, -0.03) |
| 24 weeks |  |  |  |  | -1.39 (0.3) | 0.25 (0.14, 0.45) | -0.16 (-0.26, -0.06) |
| 25 weeks |  |  |  |  | -1.28 (0.21) | 0.28 (0.18, 0.42) | -0.14 (-0.2, -0.07) |
| 26 weeks |  |  |  |  | -1.27 (0.21) | 0.28 (0.19, 0.42) | -0.14 (-0.19, -0.09) |
| All extremely preterm | -1.31 (0.17) | 0.27 (0.19, 0.37) | -0.14 (-0.19, -0.1) |  | - | - | - |
| Age (years) | -0.07 (0.14) | 0.93 (0.71, 1.23) | -0.01 (-0.05, 0.03) |  | -0.07 (0.14) | 0.93 (0.71, 1.23) | -0.01 (-0.05, 0.03) |
| IMD ≤5^c^ | -0.04 (0.17) | 0.96 (0.69, 1.33) | -0.01 (-0.05, 0.04) |  | -0.05 (0.17) | 0.95 (0.68, 1.34) | -0.01 (-0.05, 0.04) |
| Male | 0.14 (0.16) | 1.15 (0.84, 1.56) | 0.02 (-0.02, 0.06) |  | 0.13 (0.16) | 1.13 (0.82, 1.56) | 0.02 (-0.03, 0.07) |
| Non-white ethnicity | -0.11 (0.19) | 0.89 (0.61, 1.3) | -0.02 (-0.07, 0.04) |  | -0.11 (0.19) | 0.9 (0.61, 1.32) | -0.02 (-0.07, 0.04) |
| Smoker in the house | -0.15 (0.21) | 0.86 (0.57, 1.31) | -0.02 (-0.08, 0.04) |  | -0.12 (0.22) | 0.88 (0.58, 1.35) | -0.02 (-0.09, 0.05) |
| Constant | 2.95 (0.17) | 0.95 (0.93, 0.96)^d^ |  |  | 2.95 (0.18) | 0.95 (0.93, 0.96)^d^ |  |
| ^a^Cofficient (Standard error)  ^b^95% confidence intervals  ^b^Index of multiple deprivation  ^d^Exponential of the coefficient for the regression intercept | | | | | | | |
