# Supplementary material for: Economic costs and health utility values associated with extremely preterm birth: Evidence from the EPICure2 cohort study
Source: Paediatr Perinat Epidemiol. 2022 Jul 13;36(5):696–705. doi: 10.1111/ppe.12906 (PMC9543967; doi:10.1111/ppe.12906)
Supplement: Supplementary file 8 — Table S8 [file PPE-36-696-s009.docx]

eTable 8: Predictors of HUI3 (Canada MAUF model) utility score during the age 11 years)

|  | **Model 1** | | |  | **Model 2** | | |
| --- | --- | --- | --- | --- | --- | --- | --- |
| Variable | Coef (SE)^a^ | Utility ratio (95% CI)^b^ | Utility difference (95% CI)^b^ |  | Coef (SE)^a^ | Utility ratio (95% CI)^b^ | Utility difference (95% CI)^b^ |
| Gestational age at birth |  |  |  |  |  |  |  |
| 23 weeks |  |  |  |  | -2.14 (0.59) | 0.12 (0.04, 0.38) | -0.49 (-0.88, -0.11) |
| 24 weeks |  |  |  |  | -1.76 (0.4) | 0.17 (0.08, 0.37) | -0.32 (-0.59, -0.05) |
| 25 weeks |  |  |  |  | -1.58 (0.29) | 0.21 (0.12, 0.36) | -0.26 (-0.38, -0.13) |
| 26 weeks |  |  |  |  | -1.55 (0.28) | 0.21 (0.12, 0.37) | -0.25 (-0.37, -0.12) |
| All extremely preterm | -1.64 (0.22) | 0.19 (0.13, 0.3) | -0.28 (-0.37, -0.18) |  |  |  |  |
| Age (years) | -0.15 (0.19) | 0.86 (0.59, 1.25) | -0.03 (-0.12, 0.05) |  | -0.15 (0.19) | 0.86 (0.59, 1.25) | -0.04 (-0.16, 0.09) |
| IMD ≤5^c^ | -0.38 (0.23) | 0.69 (0.44, 1.07) | -0.09 (-0.19, 0.02) |  | -0.39 (0.23) | 0.68 (0.43, 1.07) | -0.09 (-0.2, 0.02) |
| Male | 0.26 (0.21) | 1.3 (0.86, 1.97) | 0.06 (-0.04, 0.16) |  | 0.24 (0.22) | 1.27 (0.83, 1.95) | 0.05 (-0.06, 0.17) |
| Non-white | -0.03 (0.26) | 0.97 (0.58, 1.6) | -0.01 (-0.13, 0.11) |  | -0.03 (0.26) | 0.97 (0.58, 1.62) | -0.01 (-0.14, 0.12) |
| Smoker in the house | -0.02 (0.29) | 0.98 (0.56, 1.72) | 0 (-0.13, 0.13) |  | 0.02 (0.29) | 1.02 (0.58, 1.82) | 0.01 (-0.13, 0.15) |
| Constant | 2.81 (0.23) | 0.94 (0.91, 0.96)^d^ |  |  | 2.83 (0.24) | 0.94 (0.91, 0.96)^d^ |  |
| ^a^Cofficient (Standard error)  ^b^95% confidence intervals  ^b^Index of multiple deprivation  ^d^Exponential of the coefficient for the regression intercept | | | | | | | |
