# Supplementary material for: Economic costs and health utility values associated with extremely preterm birth: Evidence from the EPICure2 cohort study
Source: Paediatr Perinat Epidemiol. 2022 Jul 13;36(5):696–705. doi: 10.1111/ppe.12906 (PMC9543967; doi:10.1111/ppe.12906)
Supplement: Supplementary file 9 — Appendix S1 [file PPE-36-696-s006.doc]

**Study Number**

**Date completed**

**CONFIDENTIAL**

**Questionnaire for parents**

In this questionnaire we ask you for some information about your child’s health and development, and about you and your family. We would be very grateful if you could answer all the questions as accurately as possible. We are aware that some questions may seem familiar to you as you may have already answered some of these in the previous study when your child was 6 years old. However, some of your circumstances may have changed over the last few years and we would like to keep our records up to date with the latest information possible. We would therefore appreciate it if you could answer all the questions, even if they may seem repetitive or you feel you have answered them before. We would be very grateful for your help with this questionnaire as all of this information is very important for the study.

**All the information will be treated in the strictest confidence and will not be seen by anyone outside the study. The questionnaire will also be destroyed when we have finished with it.**

*Office use only:*

**Entry 1**

**Entry 2**

**Section A. Your child’s health**

| **A1** | **In the last year, has your child had wheezing/whistling in his/her chest?** | | | | | | | | | | | | No |  |  |
| --- | --- | --- | --- | --- | --- | --- | --- | --- | --- | --- | --- | --- | --- | --- | --- |
|  |  |  |  |  |  |  |  |  |  |  |  |  |  |  |  |
|  |  |  |  |  |  |  |  |  |  |  |  |  | Yes |  |  |
|  | If no, please go to A5. | | | | | | | |  | | | | |  |  |
|  |  | | | | | | | |  | | | | |  |  |
|  |  | | | | | | | |  | | | | |  |  |
| **A2** | **In the last year, how many attacks of wheezing has your child had?** | | | | | | | | | | | | |  |  |
|  |  | |  |  | | | | | None | | | | |  |  |
|  |  | |  |  | | | | |  | | | | |  |  |
|  |  | |  |  | | | | | 1 to 3 | | | | |  |  |
|  |  | |  |  | | | | |  | | | | |  |  |
|  |  | |  |  | | | | | 4 to 12 | | | | |  |  |
|  |  | |  |  | | | | |  | | | | |  |  |
|  |  | |  |  | | | | More than 12 | | | | | |  |  |
|  |  | | | | | | | |  | | | | |  |  |
| **A3** | **In the last year, how often, on average, has your child’s sleep been disturbed due to wheezing?** | | | | | | | | |  | | | | | |
|  |  | | | | | | | |  | | | | |  |  |
|  |  | | | |  |  | Never woken with wheezing | | | | | | |  |  |
|  |  | | | |  |  |  | | | | | | |  |  |
|  |  | | | |  |  | Less than one night per week | | | | | | |  |  |
|  |  | | | |  |  |  | | | | | | |  |  |
|  |  | | | |  |  | One or more nights per week | | | | | | |  |  |
|  |  | | | | | | | |  | | | | |  |  |
|  |  | | | | | | | |  | | | | |  |  |
| **A4** | **In the last year, has wheezing ever been severe enough to limit your child’s speech to only 1 or 2 words between breaths?** | | | | | | | | | | No | | |  |  |
|  |  |  |  |  |  |  |  |  |  |  |  |  |  |  |  |
|  |  |  |  |  |  |  |  |  |  |  | Yes | | |  |  |
|  |  | | | | | | | |  | | | | |  |  |
|  |  | | | | | | | |  | | | | |  |  |
|  |  | | | | | | | |  | | | | |  |  |
| **A5** | **Has any doctor ever said that your child has asthma?** | | | | | | | | No | | | | |  |  |
|  |  | | | | | | | |  | | | | |  |  |
|  |  | | | | | | | | Yes | | | | |  |  |
|  |  | | | | | | | |  | | | | |  |  |
|  |  | | | | | | | |  | | | | |  |  |
|  |  | | | | | | | |  | | | | |  |  |
| **A6** | **In the last year, has your child’s chest sounded wheezy during or after exercise?** | | | | | | | | | | No | | |  |  |
|  |  |  |  |  |  |  |  |  |  |  |  |  |  |  |  |
|  |  |  |  |  |  |  |  |  |  |  | Yes | | |  |  |
|  |  | | | | | | | |  | | | | |  |  |
|  |  | | | | | | | |  | | | | |  |  |
|  |  | | | | | | | |  | | | | |  |  |
| **A7** | **In the last year, has your child had a dry cough at night, apart from a cough associated with a cold or a chest infection?** | | | | | | | | | | | No | |  |  |
|  |  |  |  |  |  |  |  |  |  |  |  |  |  |  |  |
|  |  |  |  |  |  |  |  |  |  |  |  | Yes | |  |  |
|  |  | | | | | | | | | | |  | |  |  |
|  |  | | | | | | | | | | |  | |  |  |
| **A8** | **In the last year, has your child seen a paediatrician or chest specialist about a chest or breathing problem?** | | | | | | | | | | | No | |  |  |
|  |  |  |  |  |  |  |  |  |  |  |  |  |  |  |  |
|  |  |  |  |  |  |  |  |  |  |  |  | Yes | |  |  |
|  |  | | | | | | | | | | |  | |  |  |
|  |  | | | | | | | | | | |  | |  |  |
|  | If yes, may we contact him/her for further information? | | | | | | | | | | | No | |  |  |
|  |  | | | | | | | | | | |  | |  |  |
|  |  |  | | | | | | | | | | Yes | |  |  |
|  | Who? |  | | | | | | | | | |  | |  |  |
|  |  |  | | | | | | | | | |  | |  |  |
|  | Where? |  | | | | | | | | | |  | |  |  |

|  | |  | | | | | | | | | | | | | | | | | | | | | |  | | | | | | | | | | |  |  | |  | | | | | | | | | |
| --- | --- | --- | --- | --- | --- | --- | --- | --- | --- | --- | --- | --- | --- | --- | --- | --- | --- | --- | --- | --- | --- | --- | --- | --- | --- | --- | --- | --- | --- | --- | --- | --- | --- | --- | --- | --- | --- | --- | --- | --- | --- | --- | --- | --- | --- | --- | --- |
| **A9** | | **In the last year, has your child been treated for any respiratory or chest problems? If yes, please give details of the type of medication (by ticking the relevant box) and giving the dose (if known) below:** | | | | | | | | | | | | | | | | | | | | | No | | |  | **If no, go to question A10** | | | | | | | | | | | | | |  | | | | | | |
|  |  |  |  |  |  |  |  |  |  |  |  |  |  |  |  |  |  |  |  |  |  |  |  |  |  |  |  |  |  |  |  |  |  |  |  |  |  |  |  |  |  |  |  |  |  |  |  |
|  |  |  |  |  |  |  |  |  |  |  |  |  |  |  |  |  |  |  |  |  |  |  | Yes | | |  | **If yes, please answer the questions a & b below:** | | | | | | | | | | | | | |  | | | | | | |
|  |  |  |  |  |  |  |  |  |  |  |  |  |  |  |  |  |  |  |  |  |  |  |  |  |  |  |  |  |  |  |  |  |  |  |  |  |  |  |  |  |  |  |  |  |  |  |  |
|  | |  | | | | | | |  | |  | | | | | |  | | | |  | | |  | | | | | | | | | | | | | |  | | | | | | | | | |
| a) Some drugs are given only when a child has breathing problems. Please tell us if your child has received any of the drugs listed below by ticking the appropriate box. If possible, please also tell us how many times they have been used in the last year. | | | | | | | | | | | | | | | | | | | | | | | | | | | | | | | | | | | | | |  | | | | | | | | | |
|  | |  | | | | **No** | | | |  | | **Yes** | | |  | | | | | | | **If yes, please give number of times** | | | | | |  | | | | | | | | |  | | | | | | | | | | |
|  | |  | | |  | |  |  | | | | |  |  | | | | | | | | | |  | | | | | | | | | | |  |  | |  | | | | | | | | | |
|  | | Antibiotics | | | | |  |  | | | | |  |  | | | | | | | | | |  | | |  | | | | | | | | | | | | | | | | | | | | |
|  | |  | | | | |  |  | | | | |  |  | | | | | | | | | |  | | |  | | |  | |  | | | | | | | | | | | | | | | |
|  | Prednisolone (Steroid tablets) | | | | | |  |  | | | | |  |  | | | | | | | | | |  | | |  | | | | | | | | | | | | | | | | | | | | |
|  | |  | | | | |  |  | | | | |  |  | | | | | | | | | |  | | |  | | |  | |  | | | | | | | | | | | | | | | |
|  | | Oxygen | | | | |  |  | | | | |  |  | | | | | | | | | |  | | |  | | | | | | | | | | | | | | | | | | | | |
|  | | Inhalers: | | |  | |  |  | | | | |  |  | | | | | |  | | | | | | | | |  | |  | |  | | | | | | | | | | | | | | |
|  | | Ventolin (blue) | | | | |  |  | | | | |  |  | | | | | | | | | |  | | |  | | | | | | | | | | | | | | | | | | | | |
|  | |  | | |  | |  |  | | | | |  |  | | | | | |  | | | | | | | | |  | |  | |  | | | | | | | | | | | | | | |
|  | | Bricanyl (blue) | | | | |  |  | | | | |  |  | | | | | | | | | |  | | |  | | | | | | | | | | | | | | | | | | | | |
|  | |  | | |  | |  |  | | | | |  |  | | | | | |  | | | | | | | | |  | |  | |  | | | | | | | | | | | | | | |
|  | | Atrovent (green) | | | | |  |  | | | | |  |  | | | | | | | | | |  | | |  | | | | | | | | | | | | | | | | | | | | |
|  | |  | | |  | |  |  | | | | |  |  | | | | | |  | | | | | | | | |  | |  | |  | | | | | | | | | | | | | | |
|  | | Salmeterol (green) | | | | |  |  | | | | |  |  | | | | | | | | | |  | | |  | | | | | | | | | | | | | | | | | | | | |
|  | |  | | |  | |  |  | | | | | |  | | | | | | |  | | |  | | | | | | | | | | |  |  | |  | | | | | | | | | |
| b) Other drugs are given to prevent symptoms / illness. Please tell us if your child has received any of these (by ticking the appropriate box) and, if possible, please tell us the dose, how often s/he takes the drug, and for how many months s/he has taken the drug in the last year. | | | | | | | | | | | | | | | | | | | | | | | | | | | | | | | | | | | | | |  | | | | | | | | | |
|  | |  | | |  |  | | | |  | |  | | |  | | | |  | | | | | | | | | | | | | | | | | | |  | | | | | | | | | |
|  | |  | | |  |  | | | |  | |  | | |  | | | |  | | | | | | | | | | | | | | | | | | |  | | | | | | | | | |
|  | |  | | |  | **No** | | | |  | | **Yes** | | |  | | | **Dose** | | | | | |  | **Number of times per day** | | | | |  | | **Number of months of treatment** | | | | | |  | | |  | | | | | | |
|  | | Inhalers: | | |  | |  |  | | | | |  |  | | | |  | | | | | |  | |  | |  | | | | | |  | |  | | | |  | | | | | | | |
|  | |  | Becotide (brown) | | | |  |  | | | | |  |  | | | |  | | | | | |  | |  | |  | | | | | |  | | |  | | |  | | | | | | | |
|  | |  | | |  | |  |  | | | | |  |  | | | |  | | | | | |  | |  | |  | | | | | |  | |  | | | |  | | | | | | | |
|  | |  | Pulmicort (brown) | | | |  |  | | | | |  |  | | | |  | | | | | |  | |  | |  | | | | | |  | | |  | | |  | | | | | | | |
|  | |  |  | | | |  |  | | | | |  |  | | | |  | | | | | |  | |  | |  | | | | | |  | |  | | | |  | | | | | | | |
|  | |  | Flixotide (orange) | | | |  |  | | | | |  |  | | | |  | | | | | |  | |  | |  | | | | | |  | | |  | | |  | | | | | | | |
|  | |  | |  | | |  |  | | | | | |  | |  | | | | | | | |  | | | | | | | | | | | | | |  | | | | | | | | | |
|  | | Please give details of any other medicines not listed above: | | | | | | | | | | | | | | | | | | | | | | | | | | | | | | | | | | | |  | |  | |  |  |  |  |  |  |
|  | |  | | |  |  | | | |  | |  | | |  | | | **Dose** | | | | | |  | **Number of times per day** | | | | |  | | **Number of months of treatment** | | | | | |  | | |  | | | | | | |
|  | |  | | |  | |  |  | | | | |  |  | | | |  | | | | | |  | |  | |  | | | | | | |  |  | | | |  | | | | | | | |
|  | | *Name:* | | | | | | | | | | | | | | | |  | | | | | |  | |  | |  | | | | | |  | | |  | | |  | | | | | | | |
|  | |  |  | | | |  |  | | | | |  |  | | | |  | | | | | |  | |  | |  | | | | | | |  |  | | | |  | | | | | | | |
|  | | *Name:* | | | | | | | | | | | | | | | |  | | | | | |  | |  | |  | | | | | |  | | |  | | |  | | | | | | | |
|  | |  |  | | | |  |  | | | | |  |  | | | |  | | | | | |  | |  | |  | | | | | | |  |  | | | |  | | | | | | | |
|  | | *Name:* | | | | | | | | | | | | | | | |  | | | | | |  | |  | |  | | | | | |  | | |  | | |  | | | | | | | |
|  | |  | |  | | |  |  | | | | | |  | |  | | | | | | | |  | | | | | | | | | | | | | |  | | | | | | | | | |

| **A10** | **Is your child up to date with his/her immunisations?** | No |  |
| --- | --- | --- | --- |
|  |  |  |  |
|  | If no, please specify the reason: | Yes |  |
|  |  |  |  |
|  |  |  |  |

| **A11** | | **a) In the last year, has your child had a fit or seizure?** | | | | | | | | | | | | | | | | | | | | | | | | | | | No | | | | | | | | | |  | | |  | |
| --- | --- | --- | --- | --- | --- | --- | --- | --- | --- | --- | --- | --- | --- | --- | --- | --- | --- | --- | --- | --- | --- | --- | --- | --- | --- | --- | --- | --- | --- | --- | --- | --- | --- | --- | --- | --- | --- | --- | --- | --- | --- | --- | --- |
|  | |  | | | | | | | | | | | | | | | | | | | | | | | | | | |  | | | | | | | | | |  | | |  | |
|  | | If no, go to A12. | | | | | | | | | | | | | | | | | | | | | | | | | | | Yes | | | | | | | | | |  | | |  | |
|  | | If yes, please answer the questions below. | | | | | | | | | | | | | | | | | | | | | | | | | | |  | | | | | | | | | |  | | |  | |
|  | |  | | | | | | | | | | | | | | | | | | | | | | | | | | |  | | | | | | | | | |  | | |  | |
|  | | **b) When was your child’s last fit or seizure?** | | | | | | | | | | | | | | | | | | | | | In last 1 month | | | | | | | | | | | | | | | |  | | |  | |
|  | |  | | | | | | | | | | | | | | | | | | | | |  | | | | | | | | | | | | | | | |  | | |  | |
|  | |  | | | | | | | | | | | | | | | | | | | | | In last 6 months | | | | | | | | | | | | | | | |  | | |  | |
|  | |  | | | | | | | | | | | | | | | | | | | | |  | | | | | | | | | | | | | | | |  | | |  | |
|  |  | | | | | | | | | | | | | | | | | | | | | | In last 12 months | | | | | | | | | | | | | | | |  | | |  | |
|  | |  | | | | | | | | | | | | | | | | | | | | | | | | | | | | |  | | | | | | | |  | | |  | |
|  | | **c) Have you been given regular medicine to control your child’s fits?** | | | | | | | | | | | | | | | | | | | | | | | | | | | | | No | | | | | | | |  | | |  | |
|  | |  | | | |  |  | | | | | | |  | |  | | | | | |  | | | | |  | |  | | | | | | | | | |  | | |  | |
|  | |  | | | | | | | | | | | | | | | | | | | |  | | | | |  | | Yes | | | | | | | | | |  | | |  | |
|  | |  | | | | | | | | | | | | | | | | | | | | | | | | | | | | | | | | | | | | | | | | | |
| If yes, please tell us the name of the medication and, if possible, please tell us the dose, how often s/he takes the drugs and for how many months s/he has taken the drug in the last year. | | | | | | | | | | | | | | | | | | | | | | | | | | | | | | | | | | | | | | | | | | | |
|  | |  | | |  | | |  | | |  |  | | | | |  | | | **Dose** | | | | |  | **Number of times per day** | | | | | | |  | | **Number of months of treatment** | | | | | | | | |
|  | |  | | |  | | | |  |  | | |  | |  | | | | |  | | | | |  | |  | | |  | | | | | | | |  | | |  | | |
| ***E.g.*** | | *Name****: Carbamazapine*** | | | | | | | | | | | | | | | | |  | ***250 mg*** | | | | |  | | ***4*** | | | | |  | | | | | ***3*** | | | | | |  |
|  | |  |  | | | | | |  |  | | |  | |  | | | | |  |  | | |  | | | |  | | | | | |  | |  | | | | | | | |
|  | | Name: | | | | | | | | | | | | | | | | |  |  | | | | |  | |  | | | | |  | | | | |  | | | | | |  |
|  | |  |  | | | | | |  |  | | |  | |  | | | | |  |  | | |  | | | |  | | | | | |  | |  | | | | | | | |
|  | | Name: | | | | | | | | | | | | | | | | |  |  | | | | |  | |  | | | | |  | | | | |  | | | | | |  |
|  | |  |  | | | | | |  |  | | |  | |  | | | | |  |  | | |  | | | |  | | | | | |  | |  | | | | | | | |
|  | | Name: | | | | | | | | | | | | | | | | |  |  | | | | |  | |  | | | | |  | | | | |  | | | | | |  |
|  | |  |  | | | | | |  |  | | |  | |  | | | | |  | | | | |  | |  | | |  | | | | | | | |  | |  | | | |
|  | |  | |  | | | | |  |  | | | | |  | | |  | | | | | | |  | | | | | | | | | | | | | | | | | | |

| **A12** | | **Is your child on any other medicines at the moment?** | | | | | | | | | | | | | | | | | | | No | | | |  | **If no, go to question A13** | | | | | | | | | | |
| --- | --- | --- | --- | --- | --- | --- | --- | --- | --- | --- | --- | --- | --- | --- | --- | --- | --- | --- | --- | --- | --- | --- | --- | --- | --- | --- | --- | --- | --- | --- | --- | --- | --- | --- | --- | --- |
|  | |  | | |  | | | | | | | |  | |  | | | | | |  | | | |  |  | | | | | | | | | |  |
|  | |  | | |  | | | | | | | |  | |  | | | | | | Yes | | | |  | **If yes, please answer the questions below:** | | | | | | | | | |  |
|  |  | | | | | | | | | | | | | | | | | | | | | | | | | | | | | | | | | |  | |
| If yes, please tell us the name of the medication and, if possible, please tell us the dose, how often s/he takes the drugs and for how many months s/he has taken the drug in the last year. | | | | | | | | | | | | | | | | | | | | | | | | | | | | | | | | | | |  | |
|  |  | | |  | |  | | |  |  | | | |  | | | **Dose** | | |  | | **Number of times per day** | | | | | |  | | **Duration of treatment (months or days)** | | | | |  | |
|  |  | | |  | | |  |  | | |  |  | | | | |  | | |  | | |  | | | |  | | | | |  | |  | | |
| ***E.g.*** | *Name:* ***Amoxycillin*** | | | | | | | | | | | | | | |  | ***250 mg*** | | |  | | | ***3*** | | | |  | | | | | ***7 days*** | | | |  |
|  |  | |  | | | |  |  | | |  |  | | | | |  |  |  | | | | |  | | | | |  | |  | | | | | |
|  | Name: | | | | | | | | | | | | | | |  |  | | |  | | |  | | | |  | | | | |  | | | |  |
|  |  | |  | | | |  |  | | |  |  | | | | |  |  |  | | | | |  | | | | |  | |  | | | | | |
|  | Name: | | | | | | | | | | | | | | |  |  | | |  | | |  | | | |  | | | | |  | | | |  |
|  |  | |  | | | |  |  | | |  |  | | | | |  |  |  | | | | |  | | | | |  | |  | | | | | |
|  | Name: | | | | | | | | | | | | | | |  |  | | |  | | |  | | | |  | | | | |  | | | |  |
|  |  | |  | | | |  |  | | |  |  | | | | |  | | |  | | |  | | | |  | | | | | |  |  | | |

*Please continue on the next page*

|  |  | | | | | | | | | | | | | | | |  | | | | |  |
| --- | --- | --- | --- | --- | --- | --- | --- | --- | --- | --- | --- | --- | --- | --- | --- | --- | --- | --- | --- | --- | --- | --- |
| **A13** | **a) Does your child have any problems with his/her hearing?** | | | | | | | | | | | | | | | | No | | | | |  |
|  |  |  |  | | |  | | | | |  |  | |  | | |  | | | | |  |
|  | If yes, please describe: | | | | | | | | | | | | | | | | Yes | | | | |  |
|  |  |  |  | | |  | | | | |  |  | |  | | |  | | | | |  |
|  |  |  |  | | |  | | | | |  |  | |  | | |  | | | | |  |
|  |  |  |  | | |  | | | | |  |  | |  | | |  | | | | |  |
|  |  |  |  | | |  | | | | |  |  | |  | | |  | | | | |  |
|  |  | | | | | | | | |  | | | | | | |  | | | | |  |
|  |  | | | | | | | | |  | | | | | | |  | | | | |  |
|  |  | | | | | | | | |  | | | | | | |  | | | | |  |
|  | **b)** **Does your child have any problems with his/her vision?** | | | | | | | | | | | | | | | | No | | | | |  |
|  |  |  |  | | |  | | | | |  |  | |  | | |  | | | | |  |
|  | If yes, please describe: | | | | | | | | | | | | | | | | Yes | | | | |  |
|  |  |  |  | | |  | | | | |  |  | |  | | |  | |  | | |  |
|  |  |  |  | | |  | | | | |  |  | |  | | |  | |  | | |  |
|  |  |  |  | | |  | | | | |  |  | |  | | |  | |  | | |  |
|  |  |  |  | | |  | | | | |  |  | |  | | |  | |  | | |  |
|  |  | | | | | | | | |  | | | | | | |  | |  | | |  |
|  |  | | | | | | | | | | | | | | | |  | | | | |  |
|  |  | | | | | | | | | | | | | | | |  | | | | |  |
|  | **c)** **Does your child have any problems with his/her speech?** | | | | | | | | | | | | | | | | No | | | | |  |
|  |  |  |  |  |  | | |  |  | | | |  | |  |  | | | |  |  | |
|  | If yes, please describe: | | | | | | | | | | | | | | | | Yes | | | | |  |
|  |  |  |  | | |  |  | | | | |  | |  | | |  | |  | | |  |
|  |  |  |  | | |  |  | | | | |  | |  | | |  | |  | | |  |
|  |  |  |  | | |  |  | | | | |  | |  | | |  | |  | | |  |
|  |  |  |  | | |  |  | | | | |  | |  | | |  | |  | | |  |
|  |  |  |  | | |  |  | | | | |  | |  | | |  | |  | | |  |
|  |  | | | | | | | | | | | | | | | | |  |  | | |  |

| **A14** | **In the last year, has your child used any of the following services?** | | | | | | | | | | | | | | | | | | | |  | | |  | |  | |  |
| --- | --- | --- | --- | --- | --- | --- | --- | --- | --- | --- | --- | --- | --- | --- | --- | --- | --- | --- | --- | --- | --- | --- | --- | --- | --- | --- | --- | --- |
|  |  | | | | | | | | | | | | | | | | | | | |  | | |  | |  | |  |
|  |  |  |  | | |  | |  | | | |  | | | | |  |  | | | | | |  | |  | |  |
|  |  |  |  | | |  | |  | | | | **Total No. of attendances** | | | | |  | **Average duration per visit (in hours)** | | | | | |  | |  | |  |
|  |  |  |  | | |  | |  | | | |  |  | |  | |  |
|  | Accident and Emergency | | | No | |  | |  | Yes |  |  | |  | |  | | | |  | | | |  |  | |  | | |
|  |  | | |  | |  | |  |  |  |  | |  | |  | | | |  | | | |  |  | |  | | |
|  | Hospital Day Unit | | | No | |  | |  | Yes |  |  | |  | |  | | | |  | | | |  |  | |  | | |
|  |  | | |  | |  | |  |  |  |  | |  | |  | | | |  | | | |  |  | |  | | |
|  | Hospital Outpatients | | | No | |  | |  | Yes |  |  | |  | |  | | | |  | | | |  |  | |  | | |
|  |  | | |  | |  | |  | |  |  | | |  | |  | | | |  | |  | | |  | |  | |
|  | If you ticked yes to any of the above please give details: | | | |  | |  | | |  |  | | |  | |  | | | |  | |  | | |  | |  | |
|  |  | |  | | |  |  | | |  | |  | | | |  | |  | | |  | |  | |
|  |  | |  | | |  |  | | |  | |  | | | |  | |  | | |  | |  | |

| **A15** | **a) In the last year, has your child been admitted to hospital for breathing difficulties? (E.g. Asthma, wheezing, respiratory infection)** | | | | | | | | | | | | | | | | | | | | | | | | | | | | | | No | | | | |  |  |
| --- | --- | --- | --- | --- | --- | --- | --- | --- | --- | --- | --- | --- | --- | --- | --- | --- | --- | --- | --- | --- | --- | --- | --- | --- | --- | --- | --- | --- | --- | --- | --- | --- | --- | --- | --- | --- | --- |
|  |  |
|  | | If yes, please specify as follows: | | | | | | | | | | | | | | | | | | | | | | Yes | | | | | | | | | | | |  |  |
|  | | |  | | | | | | |  | | | |  | | | | |  | | | | | | |  |  | | | | | | | | | | |
|  | | | | **Age** | | | **Condition** | | | | | | **Hospital** | | | | | | **Type of ward**  **eg. PICU,**  **Paediatric)** | | | | | | | | |  | | | | | **Approx**  **stay**  **(in days)** | | | | |
| Admission 1 | | | | |  |  | | |  | | | |  | |  | | |  | |  | | | | | | | | | |  | | |  | | | | |
| Admission 2 | | | | |  |  | | |  | | | |  | |  | | |  | |  | | | | | | | | | |  | | |  | | | | |
| Admission 3 | | | | |  |  | | |  | | | |  | |  | | |  | |  | | | | | | | | | |  | | |  | | | | |
| Admission 4 | | | | |  |  | | |  | | | |  | |  | | |  | |  | | | | | | | | | |  | | |  | | | | |
|  | | |  | | | | | | | | | | | | | | | | | | | | | |  | | | |  | | |  | |  | | | |
|  | | |  | | | | | | | | | | | | | | | | | | | | | |  | | | |  | | |  | |  | | | |
|  | | |  | | | | | | | | | | | | | | | | | | | | | |  | | | |  | | |  | |  | | | |
|  | | |  | | | | | | | | | | | | | | | | | | | | | |  | | | |  | | |  | |  | | | |
|  | | | b) In the last year, has your child been admitted to hospital for surgery? | | | | | | | | | | | | | | | | | | | | | | No | | | | | | | | | | |  |  |
|  | | |  | | | | |  | | |  |  | | | |  |  | | |  | | |  | | | |  | | | | | |  | |  | | |
|  | | | If yes, please specify as follows: | | | | | | | | | | | | |  |  | | |  | | Yes | | | | | | | | | | | | | |  |  |
|  | | |  | | | | | | |  | | | |  | | | | |  | | | | | | |  |  | | | | | | | | | | |
|  | | | **Age** | | | | | | | **Condition** | | | | **Hospital** | | | | | **Type of ward**  **eg. PICU,**  **Paediatric)** | | | | | | | | |  | | | | | **Approx**  **stay**  **(in days)** | | | | |
| Admission 1 | | | | |  | | | | | | | | | | | | | | | | | |  | | | | | | | | | |  | | | | |
| Admission 2 | | | | |  |  | | |  | | | |  | |  | | |  | |  | | | | | | | | | |  | | |  | | | | |
| Admission 3 | | | | |  |  | | |  | | | |  | |  | | |  | |  | | | | | | | | | |  | | |  | | | | |
| Admission 4 | | | | |  |  | | |  | | | |  | |  | | |  | |  | | | | | | | | | |  | | |  | | | | |
|  | | |  | | | | | | | | | | | | | | | | | |  | | | | | | | |  | | |  | |  | | | |

|  | **c) In the last year, has your child been admitted to hospital for any other reasons? E.g. Fits, gastroenteritis** | | | | | | | | | | | | | | | | | | | | | | | | | | | No | | | |  | |  |
| --- | --- | --- | --- | --- | --- | --- | --- | --- | --- | --- | --- | --- | --- | --- | --- | --- | --- | --- | --- | --- | --- | --- | --- | --- | --- | --- | --- | --- | --- | --- | --- | --- | --- | --- |
|  |  |  |  |  |  |  |  |  |  |  |  |  |  |  |  |  |  |  |  |  |  |  |  |  |  |  |  |  |  |  |  |  |  |  |
|  |  |  |  |  |  |  |  |  |  |  |  |  |  |  |  |  |  |  |  |  |  |  |  |  |  |  |  | Yes | | | |  | |  |
|  | If yes, please specify as follows: | | | | | | | | | | | |  |  | | |  | |  | | | | | |  | |  | | | | | | | |
|  |  | | | | |  | | | |  | | | | | |  | | | | |  | |  | | | | | | | | | | | |
|  | **Age** | | | | | **Condition** | | | | **Hospital** | | | | | | **Type of ward**  **eg. PICU,**  **Paediatric)** | | | | | | | |  | | | | **Approx**  **stay**  **(in days)** | | | | | | |
| Admission 1 | |  | | | | | | | | | | | | | | | | | |  | | | | | | | |  | | | | | | |
| Admission 2 | |  |  | |  | | | |  | | |  | | |  | |  | | | | | | | | |  | | |  | | | | | |
| Admission 3 | |  |  | |  | | | |  | | |  | | |  | |  | | | | | | | | |  | | |  | | | | | |
| Admission 4 | |  |  | |  | | | |  | | |  | | |  | |  | | | | | | | | |  | | |  | | | | | |
|  |  | | |  | | |  |  | | |  | | |  | | |  |  | | | |  | | | | | |  | | |  | | | |

|  |  | | |  | | |  |  | | |  | | |  | | |  |  | | | |  | | | | | | |  | | |  | | | |
| --- | --- | --- | --- | --- | --- | --- | --- | --- | --- | --- | --- | --- | --- | --- | --- | --- | --- | --- | --- | --- | --- | --- | --- | --- | --- | --- | --- | --- | --- | --- | --- | --- | --- | --- | --- |
|  |  | | |  | | |  |  | | |  | | |  | | |  |  | | | |  | | | | | | |  | | |  | | | |
|  | **d) In the last year, has your child been admitted to an intensive care unit?** | | | | | | | | | | | | | | | | | | | | | | | No | | | | | | | | |  | |  |
|  |  |  |  |  |  |  |  |  |  |  |  |  |  |  |  |  |  |  |  |  |  |  |  |  |  |  |  |  |  |  |  |  |  |  |  |
|  |  |  |  |  |  |  |  |  |  |  |  |  |  |  |  |  |  |  |  |  |  |  |  | Yes | | | | | | | | |  | |  |
|  | If yes, please specify as follows: | | | | | | | | | | | |  |  | | |  | |  | | | | | |  | | |  | | | | | | | |
|  |  | | | | |  | | | |  | | | | | |  | | | | |  | |  | | | | | | | | | | | | |
|  | **Age** | | | | | **Condition** | | | | **Hospital** | | | | | | **Approx stay**  **(in days)** | | | | | | | | | | | | |  | | | | | | |
| Admission 1 | |  | | | | | | | | | | | | | | | | | |  | | | | | | | | |  | | | | | | |
| Admission 2 | |  |  | |  | | | |  | | |  | | |  | |  | | | | | | | | | |  | | |  | | | | | |
| Admission 3 | |  |  | |  | | | |  | | |  | | |  | |  | | | | | | | | | |  | | |  | | | | | |

| **A16** | **a) Does your child have any long-term illness not mentioned above?** | | | | | | | | | | | | | | | | No | | | | | | |  | | |  | |  | |  | |
| --- | --- | --- | --- | --- | --- | --- | --- | --- | --- | --- | --- | --- | --- | --- | --- | --- | --- | --- | --- | --- | --- | --- | --- | --- | --- | --- | --- | --- | --- | --- | --- | --- |
|  |  | | | | | | | | | | | | |  | | | | | | | | | |  | | |  | | |  | |  |
|  | If no, go to A17. | | | | | | | | | | | | | Yes | | | | | | | | | |  | | |  | |  | |  | |
|  | If yes, please give details: | | | | | | | | | | | | |  | | | | | |  | | | | |  | | |  | | |  | |
|  |  | | | | | | | | | | | | |  | | | | |  | | |  | | | | | |  | | |  | |
|  |  | | | | | | | | | | | | |  | | | | |  | | |  | | | | | |  | | |  | |
|  |  | | | | | | | | | | | | |  | | | | |  | | |  | | | | | |  | | |  | |
|  |  | | | | | | | | | | | | |  | | | | |  | | |  | | | | | |  | | |  | |
|  | **b) Has your child ever been admitted to hospital for this illness?** | | | | | | | | | | | | | | | No | | | | | | | |  | | |  | |  | |  | |
|  |  | | | | | | | | | | | | | | |  | | | | | | | |  | | |  | |  | |  | |
|  | If no, go to A17. | | | | | | | | | | | | | | | Yes | | | | | | | |  | | |  | |  | |  | |
|  | | If yes, please give details: | | | | | | | | | | | | | | | | | | | | | | | | | | | | | | |
|  | | |  | | | |  | |  | | |  | | | | | |  | | | | | | |  | | | | | | | |
|  | | |  | | | |  | |  | | |  | | | | | |  | | | | | | |  | | | | | | | |
|  | | | **Age** | | | | **Condition** | | **Name of Hospital** | | | **Type of ward**  **(eg. PICU, Paediatric)** | | | | | | | | |  | | | | **Approx stay**  **(in days)** | | | | | | | |
| Admission 1 | | | |  | | | | | | | | | | |  | | | | | | | | | |  | | | | | | | |
| Admission 2 | | | |  |  |  | |  | |  |  | |  | | | | | | | | | |  | | |  | | | | | | |
| Admission 3 | | | |  |  |  | |  | |  |  | |  | | | | | | | | | |  | | |  | | | | | | |
| Admission 4 | | | |  |  |  | |  | |  |  | |  | | | | | | | | | |  | | |  | | | | | | |

| **A17** | | **a) Have you had to take any time off work over the last year as a** | | | | |  | No | | |  | |  | | |  |
| --- | --- | --- | --- | --- | --- | --- | --- | --- | --- | --- | --- | --- | --- | --- | --- | --- |
|  | | **result of your child’s health?** |  | |  |  | | | | |  | |  | |  | |
|  | |  |  | |  | Yes | | | | |  | |  | |  | |
|  | |  |  | |  |  | | | |  | |  | | |  | |
|  | |  |  | |  |  | | | |  | |  | | |  | |
|  | |  |  | |  | N/A | | | | |  | |  | |  | |
|  | |  |  | |  |  | | | | |  | |  | |  | |
|  |  | **b) If yes,** please estimate how many days you have had off work? | |  | | | | | days | | | | |  |  | |

| **A18** | | **a) Has your partner had to take any time off work over the last year** | | | | |  | No |  |  | |  |  |
| --- | --- | --- | --- | --- | --- | --- | --- | --- | --- | --- | --- | --- | --- |
|  | | **as a result of your child’s health?** |  | |  |  | | |  |  | |  | |
|  | |  |  | |  | Yes | | |  |  | |  | |
|  | |  |  | |  |  | | |  |  | |  | |
|  | |  |  | |  | N/A | | |  |  | |  | |
|  | |  |  | |  |  | | |  |  | |  | |
|  |  | **b) If yes,** please estimate how many days s/he has had off work? | |  | | | | days | | |  |  |  |

| **A19** | | **a) Has any other relative or friend had to take any time off work** | | | | | |  | No | |  |  | |  |  |
| --- | --- | --- | --- | --- | --- | --- | --- | --- | --- | --- | --- | --- | --- | --- | --- |
|  | | **over the last year as a result of your child’s health?** |  | |  |  | | | | |  |  | |  | |
|  | |  |  | |  | Yes | | | | |  |  | |  | |
|  | |  |  | |  |  | | | | |  |  | |  | |
|  | |  |  | |  | N/A | | | | |  |  | |  | |
|  |  |  | |  | | |  | | |  | | |  |  |  |
|  |  | **b) If yes**, please estimate how many days s/he has had off work? | |  | | | | | | Days | | |  |  |  |

| **A20** | **As a result of your child’s health, over the last year:** | |  | |  | |  | |  | |
| --- | --- | --- | --- | --- | --- | --- | --- | --- | --- | --- |
|  |  | |  | |  | |  | |  | |
|  | **a) Has anybody in the family had to turn down an employment opportunity?** | | | | | No | |  | |  |
|  |  |  |  |  |  |  |  |  |  |  |
|  |  |  |  |  |  | Yes | |  | |  |
|  |  | |  | | | | |  | |  |
|  |  |  | | | | | |  | |  |
|  | **b) Has anybody in the family had to leave existing employment?** | | | No | | | |  | |  |
|  |  |  |  |  |  |  |  |  |  |  |
|  |  |  |  | Yes | | | |  | |  |
|  |  | |  | | | | |  | |  |
|  |  |  | | | | | |  | |  |
|  | **c) Has anybody in the family felt unable to work?** | No | | | | | |  | |  |
|  |  |  |  |  |  |  |  |  |  |  |
|  |  | Yes | | | | | |  | |  |

| **A21** | **Does your child’s health affect either your work or your partner’s work now?** | | | | | | No | | | |  | |  | | |  |
| --- | --- | --- | --- | --- | --- | --- | --- | --- | --- | --- | --- | --- | --- | --- | --- | --- |
|  |  |  |  |  |  |  |  |  |  |  |  |  |  |  |  |  |
|  |  |  |  |  |  |  | Yes | | | |  | |  | | |  |
|  |  |  |  |  |  |  |  |  |  |  |  |  |  |  |  |  |
|  |  | | Not now, but was true in the past | | | | | | | |  | |  | | |  |
|  |  | |  | | |  | |  | | | |  | | |  | |
|  |  | | | | | | | | | | | | | |  | |
|  | **If you answered yes**, please mark any of the following that have been affected by your child’s health.  (You can mark more than one). | | | | | | | | | | | | | |  | |
|  |  |  |  |  |  |  |  |  |  |  |  |  |  |  |  |  |
|  | The type of work you do? | | | |  | |  | | |  | |  | | |  | |
|  |  |  | | |  | |  | | |  | |  | | |  | |
|  | The opportunity for changing jobs? | | | |  | |  | | |  | |  | | |  | |
|  |  |  | | |  | |  | | |  | |  | | |  | |
|  | The hours you work? | | | |  | |  | | |  | |  | | |  | |
|  |  |  | | |  | |  | | |  | |  | | |  | |
|  | The amount of time you are distracted/disrupted during your workday? | | | |  | |  | | |  | |  | | |  | |
|  |  |  | | |  | |  | | |  | |  | | |  | |
|  | The amount of time taken off for appointments for the education of your child? | | | |  | |  | | |  | |  | | |  | |
|  |  |  | | |  | |  | | |  | |  | | |  | |
|  | Your promotion prospects? | | | |  | |  | | |  | |  | | |  | |
|  |  |  | | |  | |  | | |  | |  | | |  | |
|  | Your ability to keep your job? | | | |  | |  | | |  | |  | | |  | |
|  |  |  | | |  | |  | | |  | |  | | |  | |
|  | The distance you can travel to work? | | | |  | |  | | |  | |  | | |  | |
|  |  |  | | |  | |  | | |  | |  | | |  | |
|  | Your attendance at work? | | | |  | |  | | |  | |  | | |  | |
|  |  |  | | |  | |  | | |  | |  | | |  | |
|  | Anything else? (Specify below) | | | |  | |  | | |  | |  | | |  | |
|  |  | | |  | | |  | |  | | | | |  | | |
|  | Please specify any other : | | |  | | |  | |  | | | | |  | | |
|  |  | | |  | | |  | |  | | | | |  | | |
|  |  | | |  | | |  | |  | | | | |  | | |

| **A22** | **Because of your child’s health, do you think your family find it difficult to make ends meet?** | No |  |  |  |
| --- | --- | --- | --- | --- | --- |
|  |  |  |  |  |  |
|  |  | Yes |  |  |  |
|  |  |  |  |  |  |

| ***This information, along with the rest of the questionnaire, is for our use only and will not be disclosed to any other party.*** |
| --- |
|

| **A23** | **Do you or anyone in your family receive any benefits at the moment?** | | | | | | | | No |  |  |  | |
| --- | --- | --- | --- | --- | --- | --- | --- | --- | --- | --- | --- | --- | --- |
|  |  |  | | | |  |  |  | |  |  | |  |
|  |  | | | | | | | Yes | |  |  | |  |
|  |  |  | |  |
|  | If yes, please indicate which benefits you receive: | | | | | | |  | |  |  | |  |
|  |  |  | | | |  |  |  | |  |  | |  |
|  | Child Benefit | |  |  |  | | | | |  |  | |  |
|  |  |  |  |  |  | | | | |  |  | |  |
|  | Income Support | |  |  |  | | | | |  |  | |  |
|  |  |  |  |  |  | | | | |  |  | |  |
|  | Family Credit | |  |  |  | | | | |  |  | |  |
|  |  |  |  |  |  | | | | |  |  | |  |
|  | Care Allowance | |  |  |  | | | | |  |  | |  |
|  |  |  |  |  |  | | | | |  |  | |  |
|  | Job Seekers Allowance | |  |  |  | | | | |  |  | |  |
|  |  |  |  |  |  | | | | |  |  | |  |
|  | Disability Living Allowance | |  |  |  | | | | |  |  | |  |
|  |  |  |  |  |  | | | | |  |  | |  |
|  | Housing Benefit | |  |  |  | | | | |  |  | |  |
|  |  |  |  |  |  | | | | |  |  | |  |
|  | Maternity Benefit | |  |  |  | | | | |  |  | |  |
|  |  |  |  |  |  | | | | |  |  | |  |
|  |  | Other |  |  | Please describe: | | | | | | | |  |

| **A24a** | **Are any of the following items costing you more because of your child’s health? If yes, please estimate the additional cost of these items over the last MONTH.** | | | | | | | | | | |  | | | | |
| --- | --- | --- | --- | --- | --- | --- | --- | --- | --- | --- | --- | --- | --- | --- | --- | --- |
|  |  | | | | |
|  | **No Yes If yes, give total additional cost**  **over the last MONTH** | | | | | | | | | | | | | | | |
|  | Food | |  |  |  |  |  |  |  | | | |  | |  | |
|  |  |  |  |  |  |  |  |  |  |  |  | | |  | |  |
|  | Bedding | |  |  |  |  |  |  |  | | | | |  | |  |
|  |  |  |  |  |  |  |  |  |  |  |  | | |  | |  |
|  | Washing / Laundry | |  |  |  |  |  |  |  | | | | |  | |  |
|  |  |  |  |  |  |  |  |  |  |  |  | | |  | |  |
|  | Clothing | |  |  |  |  |  |  |  | | | | |  | |  |
|  |  |  |  |  |  |  |  |  |  |  |  | | |  | |  |
|  | Cost of visiting hospital or GP | |  |  |  |  |  |  |  | | | | |  | |  |
|  |  |  |  |  |  |  |  |  |  |  |  | | |  | |  |
|  | Child Care | |  |  |  |  |  |  |  | | | | |  | |  |
|  |  |  |  |  |  |  |  |  |  |  |  | | |  | |  |
|  | Help with housework | |  |  |  |  |  |  |  | | | | |  | |  |
|  |  |  |  |  |  |  |  |  |  |  |  | | |  | |  |
|  | Telephone bills | |  |  |  |  |  |  |  | | | | |  | |  |
|  |  |  |  |  |  |  |  |  |  |  |  | | |  | |  |

| **A24b** | | **Are any of the following items costing you more because of your child’s health?**  **If yes, please estimate the additional cost of these items over the last YEAR.** | | | | | | | | | | | | | |  | | | |
| --- | --- | --- | --- | --- | --- | --- | --- | --- | --- | --- | --- | --- | --- | --- | --- | --- | --- | --- | --- |
|  | | **No Yes If yes, give total additional cost**  **over the last YEAR** | | | | | | | | | | | | | | | | | |
|  |  | Therapies for your child | |  |  | |  |  |  |  |  | | | | | | |  | |
|  |  |  |  |  |  | |  |  |  |  |  | |  | |  | | |  | |
|  |  | Repairs or adaptations to your home | |  |  | |  |  |  |  |  | | | | | | |  | |
|  |  |  | |  |  | |  |  |  |  |  | |  | |  | | |  | |
|  |  | Special equipment for your child | |  |  | |  |  |  |  |  | | | | | | |  | |
|  |  |  | |  |  | |  |  |  |  |  | |  | |  | | |  | |
|  | Other expense (Please specify below): below) | | |  |  | |  |  |  |  |  | | | | | | |  | |
|  |  |  |  |  |  |  | | | | | |  | |  | | |  | |  |

**Section B. Health and Social Service Use**

| Children come into contact with a number of community professionals. We would be grateful if you could indicate with a tick, which, if any, your child has seen in the last year. | | | | | | | | | | | | | | | | | | |  | | |
| --- | --- | --- | --- | --- | --- | --- | --- | --- | --- | --- | --- | --- | --- | --- | --- | --- | --- | --- | --- | --- | --- |
|  | |  | | | | | | | | | | | | |  | | |  | | | |
| **Tick if Yes & Give No of occasions** | | | | | | | | | | | | | | **OR tick if none** | | | | |  | |  |
|  |  | |  | | | |  |  | |  |  |  | |  | |  |  |  | |  |  |
|  | **1** | | **General Practitioner (GP)** | | | |  | on | |  |  | occasions | |  | |  |  |  | |  |  |
|  |  | |  | | | |  |  | |  |  |  | |  | |  |  |  | |  |  |
|  | **2** | | **Practice Nurse** | | | |  | on | |  |  | occasions | |  | |  |  |  | |  |  |
|  |  | |  | | | |  |  | |  |  |  | |  | |  |  |  | |  |  |
|  | **3** | | **Community Nurse** | | | |  | on | |  |  | occasions | |  | |  |  |  | |  |  |
|  |  | |  | | | |  |  | |  |  |  | |  | |  |  |  | |  |  |
|  | **4** | | **Community Paediatrician** | | | |  | on | |  |  | occasions | |  | |  |  |  | |  |  |
|  |  | |  | | | |  |  | |  |  |  | |  | |  |  |  | |  |  |
|  | **5** | | **Dentist** | | | |  | on | |  |  | occasions | |  | |  |  |  | |  |  |
|  |  | |  | | | |  |  | |  |  |  | |  | |  |  |  | |  |  |
|  | **6** | | **Orthodontist** | | | |  | on | |  |  | occasions | |  | |  |  |  | |  |  |
|  |  | |  | | | |  |  | |  |  |  | |  | |  |  |  | |  |  |
|  | **7** | | **Optician** | | | |  | on | |  |  | occasions | |  | |  |  |  | |  |  |
|  |  | |  | | | |  |  | |  |  |  | |  | |  |  |  | |  |  |
|  | **8** | | **Chiropodist** | | | |  | on | |  |  | occasions | |  | |  |  |  | |  |  |
|  |  | |  | | | |  |  | |  |  |  | |  | |  |  |  | |  |  |
|  | **9** | | **Physiotherapist** | | | |  | on | |  |  | occasions | |  | |  |  |  | |  |  |
|  |  | |  | | | |  |  | |  |  |  | |  | |  |  |  | |  |  |
|  | **10** | | **Speech Therapist** | | | |  | on | |  |  | occasions | |  | |  |  |  | |  |  |
|  |  | |  | | | |  |  | |  |  |  | |  | |  |  |  | |  |  |
|  | **11** | | **Audiologist (Hearing Specialist)** | | | |  | on | |  |  | occasions | |  | |  |  |  | |  |  |
|  |  | |  | | | |  |  | |  |  |  | |  | |  |  |  | |  |  |
|  | **12** | | **Social Worker** | | | |  | on | |  |  | occasions | |  | |  |  |  | |  |  |
|  |  | |  | | | |  |  | |  |  |  | |  | |  |  |  | |  |  |
|  | **13** | | **Home Visitor / Volunteer** | | | |  | on | |  |  | occasions | |  | |  |  |  | |  |  |
|  |  | |  | | | |  |  | |  |  |  | |  | |  |  |  | |  |  |
|  | **14** | | **Counsellor** | | | |  | on | |  |  | occasions | |  | |  |  |  | |  |  |
|  |  | |  | | | |  |  | |  |  |  | |  | |  |  |  | |  |  |
|  | **15** | | **Psychologist** | | | |  | on | |  |  | occasions | |  | |  |  |  | |  |  |
|  |  | |  | | | |  |  | |  |  |  | |  | |  |  |  | |  |  |
|  | **16** | | **Psychiatrist** | | | |  | on | |  |  | occasions | |  | |  |  |  | |  |  |
|  |  | |  | | | |  |  | |  |  |  | |  | |  |  |  | |  |  |
|  | **17** | | **Osteopath** | | | |  | on | |  |  | occasions | |  | |  |  |  | |  |  |
|  |  | |  | | | |  |  | |  |  |  | |  | |  |  |  | |  |  |
|  | **18** | | **Home teacher (Portage)** | | | |  | on | |  |  | occasions | |  | |  |  |  | |  |  |
|  |  | |  | | | |  |  | |  |  |  | |  | |  |  |  | |  |  |
|  | **19** | | **Home teacher (Other)** | | | |  | on | |  |  | occasions | |  | |  |  |  | |  |  |
|  |  | |  | | | |  |  | |  |  |  | |  | |  |  |  | |  |  |
|  | **20** | | **Orthoptist** | | | |  | on | |  |  | occasions | |  | |  |  |  | |  |  |
|  |  | |  | | | |  |  | |  |  |  | |  | |  |  |  | |  |  |
|  | **21** | | **Orthotist (Mobility specialist)** | | | |  | on | |  |  | occasions | |  | |  |  |  | |  |  |
|  |  | |  | | | |  |  | |  |  |  | |  | |  |  |  | |  |  |
|  | **22** | | **Other (specify)** | | | |  | on | |  |  | occasions | |  | |  |  |  | |  |  |
|  |  |  | |  |  |  | | |  | | | |  |  | |  |  |  | | |  |
|  | | | | | | | | | | | | | |  | |  |  |  | | |  |


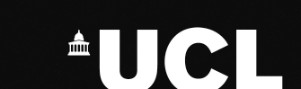


**Office Use only**


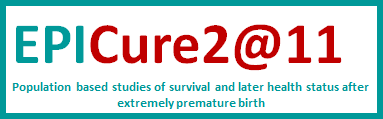


# Teacher questionnaire

**CONFIDENTIAL**

**To be completed by the class teacher or form teacher of a young person taking part in the EPICure2@11 Study**

# Instructions for completing this questionnaire

Dear teacher,

This child is taking part in the EPICure2@11 Study – a study of premature infants’ and their peers’ memory, learning, health, and development as they grow up. We have obtained permission from this child’s parent/guardian to ask you for some important information relating to his/her classroom behaviour **over the last six months** and his/her achievement during the **current academic year**. We would be very grateful if you would complete this questionnaire.

*How to complete this questionnaire:*

Sections A and B in this questionnaire ask for information about this child’s special educational needs provision, achievement, and attendance over the previous school year. You may find it helpful to contact your administrator, SENCO and/or other teachers involved in this child’s learning when filling in these sections. The remainder of the questionnaire is focused on this child’s behaviour and well-being.

**Please answer all questions** as best you can, even if you are not absolutely certain or the question doesn’t seem very relevant to this child. We will treat all the information in the **strictest confidence**. Parents will **not** have access to this information and we will not divulge it to anyone outside the study. The questionnaire will also be destroyed when we have finished with it.

**Once you have the information needed, please complete the questionnaire and post it back to us in the freepost envelope provided.**

*For further information:*

We have enclosed an information sheet with this questionnaire that tells you more about the study. If you have any questions or would like further information, please telephone the EPICure2 Study Office at University College London on 020 3108 2045 or email the study team at [epicure@ucl.ac.uk.](mailto:epicure@ucl.ac.uk) You can also visit the EPICure2 Study website at: [www.epicure.ac.uk.](http://www.epicure.ac.uk/)

**Thank you for completing this questionnaire and helping with this important study.**

**Your contribution is greatly appreciated.**

# Section A: Educational Provision

We would like to know about this child’s school attendance and provision of SEN support.

**A1 On what date did the previous school term commence?**

**day**

**month**

**year**

**A2 On what date did the previous school term end?**

**day**

**month**

**year**

**A3 In total, how many sessions were there in the previous school term (a session equals half a day)?**

**A4 In total, how many sessions did this child miss due to absence (include both authorised and unauthorised absences) in the previous term?**

**A5 Does this child have any special educational needs? yes no**

If **yes**, please specify the child’s area of need(s) from the list below.

***Please tick***

***as appropriate***

Speech, learning and communication needs

Autism Spectrum Disorder

Moderate learning difficulty

Specific learning difficulty

Behaviour or social, emotional and mental health difficulties

Hearing impairment

Visual impairment

Multi-sensory impairment

Physical disability

**A6 Does this child have a Statement of Special Educational Needs (SEN), or an Education, Health and Social Care (EHC)**

**Plan? yes no**

**A7 Does this child receive SEN support in school?**

If **YES**, please indicate the type(s) of support this child currently receives, and specify the number of hours support received per week.

Individual Education/Behaviour plan One-to-one special needs provision Small group special needs provision

**yes no**

***Please tick***

***as appropriate***

***Hours per***

***week***

Outreach teacher(s) Educational psychologist Clinical psychologist Physiotherapist

Speech therapist Occupational Therapist

**A8 In your professional opinion, does this child need extra support in addition to that which is currently provided, if any)?**

If **YES**, please indicate the type(s) of support you feel this child needs.

Individual Education/Behaviour plan One-to-one special needs provision Small group special needs provision

**yes no**

***Please tick as appropriate***

Outreach teacher(s) Educational psychologist Clinical psychologist Physiotherapist

Speech therapist Occupational Therapist

**Completed by:**

**Name (CAPITALS) _ _ _ _ _ _ _ _ _ _ _ _ _ _ _ _ _ _ _ _ _ _ _ _ _ _ _ _ _ _ _ _ _ _ _ _ _ _ _ _ _ _ _ _ _**

**Signature _ _ _ _ _ _ _ _ _ _ _ _ _ _ _ _ _ _ _ _ _ _ _ _ _ _ _ _ _ _ _ _ _ _ _ _ _ _ _ _ _ _ _ _ _ _ _ _ _**

**Date _ _ _ _ _ _ _ _ _ _ _ _ _ _ _ _ _ _ _ _ _ _ _ _ _ _ _ _ _ _ _ _ _ _ _ _ _ _ _ _ _ _ _ _ _ _ _ _ _ _ _ _**

**Present Class Teacher / Previous Class Teacher / Form Teacher / Other *(Please circle)***

**Thank you very much for your time and help with this important study**
